# NLRP inflammasomes in health and disease

**DOI:** 10.1186/s43556-024-00179-x

**Published:** 2024-04-22

**Authors:** Zhihao Xu, Arnaud John Kombe Kombe, Shasha Deng, Hongliang Zhang, Songquan Wu, Jianbin Ruan, Ying Zhou, Tengchuan Jin

**Affiliations:** 1 Center of Disease Immunity and Intervention, College of Medicine, Lishui University, Lishui, 323000 China; 2 Laboratory of Structural Immunology, the CAS Key Laboratory of Innate Immunity and Chronic Disease, School of Basic Medical Sciences, Division of Life Sciences and Medicine, University of Science and Technology of China, Hefei, 230027 China; 3 Department of Immunology, University of Connecticut Health Center, Farmington, 06030 USA; 4 Department of Obstetrics and Gynecology, Core Facility Center, Division of Life Sciences and Medicine, The First Affiliated Hospital of USTC, University of Science and Technology of China, Hefei, 230001 Anhui China; 5 Institute of Health and Medicine, Hefei Comprehensive National Science Center, Hefei, Anhui China; 6 Biomedical Sciences and Health Laboratory of Anhui Province, University of Science & Technology of China, Hefei, 230027 China; 7 Clinical Research Hospital of Chinese Academy of Sciences (Hefei), University of Science and Technology of China, Hefei, 230001 China

**Keywords:** NLRP inflammasome, Health and disease, Therapeutic inhibitor, Auto-inflammatory, Autoimmune, Neurological disorders

## Abstract

NLRP inflammasomes are a group of cytosolic multiprotein oligomer pattern recognition receptors (PRRs) involved in the recognition of pathogen-associated molecular patterns (PAMPs) and danger-associated molecular patterns (DAMPs) produced by infected cells. They regulate innate immunity by triggering a protective inflammatory response. However, despite their protective role, aberrant NLPR inflammasome activation and gain-of-function mutations in NLRP sensor proteins are involved in occurrence and enhancement of non-communicating autoimmune, auto-inflammatory, and neurodegenerative diseases. In the last few years, significant advances have been achieved in the understanding of the NLRP inflammasome physiological functions and their molecular mechanisms of activation, as well as therapeutics that target NLRP inflammasome activity in inflammatory diseases. Here, we provide the latest research progress on NLRP inflammasomes, including NLRP1, CARD8, NLRP3, NLRP6, NLRP7, NLRP2, NLRP9, NLRP10, and NLRP12 regarding their structural and assembling features, signaling transduction and molecular activation mechanisms. Importantly, we highlight the mechanisms associated with NLRP inflammasome dysregulation involved in numerous human auto-inflammatory, autoimmune, and neurodegenerative diseases. Overall, we summarize the latest discoveries in NLRP biology, their forming inflammasomes, and their role in health and diseases, and provide therapeutic strategies and perspectives for future studies about NLRP inflammasomes.

## Introduction

The host's innate immune system contributes to recognizing and responding to cellular stress and danger signals [1, 2]. Pattern recognition receptors (PRRs) of the innate immune system mediate recognition of conserved molecular signatures of pathogen-associated molecular patterns (PAMPs) and damage-associated molecular patterns (DAMPs) [2, 3]. PRRs are usually classified into five main classes by different receptor proteins, including Toll-like receptors (TLRs), C-type lectin receptors (CLRs), RIG-I-like receptors (RLRs), absent in melanoma 2 (AIM2)-like receptors (ALRs) and nucleotide-binding domain and leucine-rich repeat receptors (NLRs) [2, 4]. TLRs and CLRs are transmembrane proteins that recognize extracellular PAMPs and DAMPs or within endosomes. The other cited protein receptors, including RLRs, ALRs and NLRs, are thought to detect cytosolic or intracellular PAMPs and DAMPs. Among these receptors, certain NLRs and ALRs can assemble into high-weight oligomeric complexes known as inflammasomes. The term inflammasome was first used by Tschopp and colleagues two decades ago [2, 5].

Inflammasomes are a set of cytoplasmic receptor proteins usually triggered in response to cellular stress associated with infectious agents and physiological aberration. Inflammasomes typically comprise a cytosolic NLR or ALR sensor, an adaptor ASC (apoptosis-associated speck-like protein containing a caspase activation and recruitment domain, CARD), and a cysteine protease caspase-1 [6–8]. Based on the different protein components and activation pathways, inflammasomes were traditionally categorized into two main groups: canonical and non-canonical inflammasomes [2, 9, 10]. Canonical inflammasomes were found earlier to form a sensor-ASC-caspase-1 platform for inflammatory caspase-1 activation. Their multiprotein complex formation depends on different cytosolic sensors, mostly from NLR members, ALR members, including AIM2, or the tripartite motif (TRIM) family member, like pyrin [2, 11, 12]. The non-canonical inflammasomes that assemble without dedicated PRRs have similar functions as canonical inflammasomes response to lipopolysaccharide (LPS) and cell endogenous oxidized lipids (oxPAC) by the activation of caspase-11 in mice and caspase-4 and -5 in human [9, 10, 13, 14]. In addition, inflammasomes are widely characterized as protein complexes of activation of inflammatory caspase-1 and a regulated form of cell death called pyroptosis accompanied by DNA fragmentation and rapid plasma membrane permeability [2, 15–18]

**Fig. 1:**
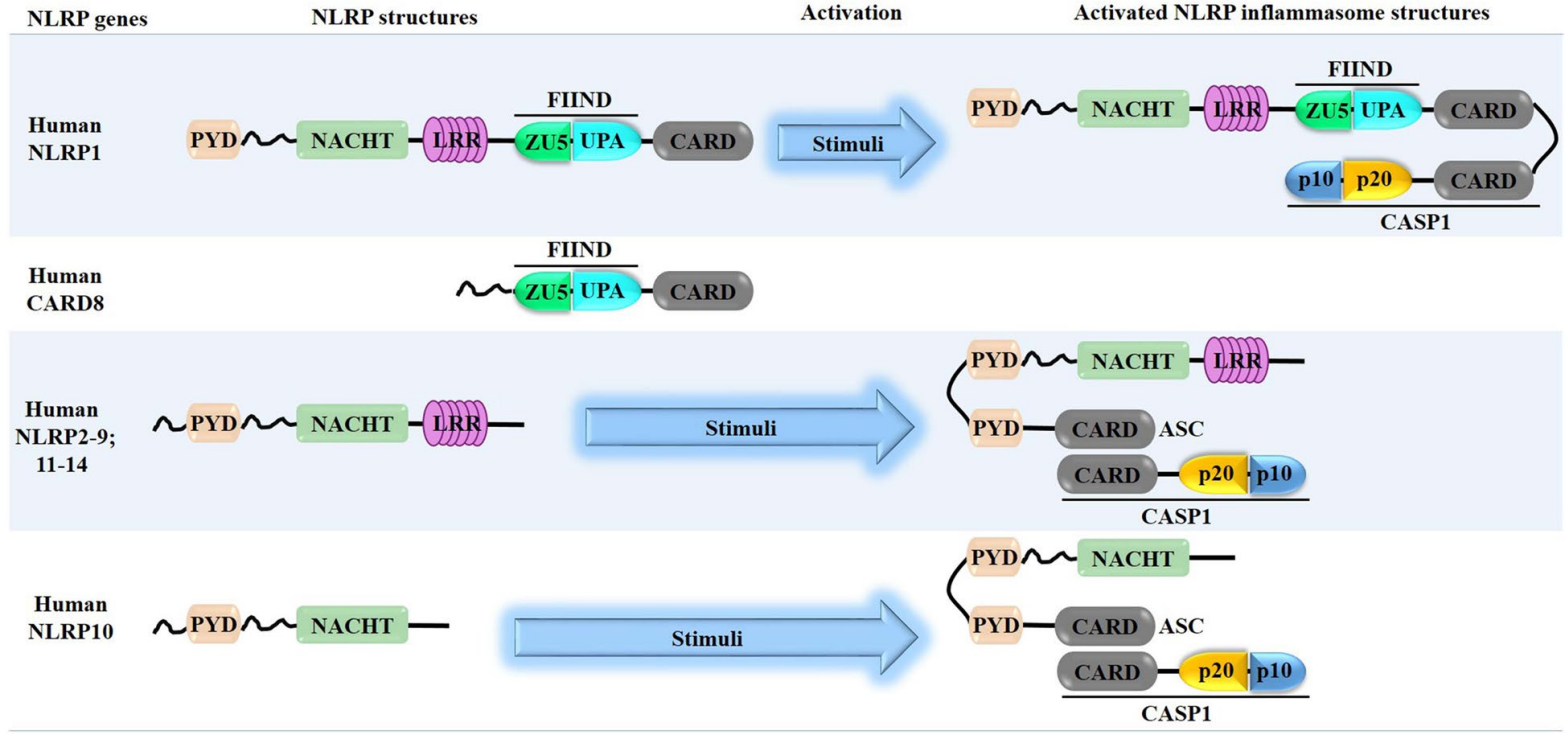
Overview of Structural organization of NLRP family and NLRP inflammasome assembly. NLRP family genes consist of an N-terminal pyrin domain (PYD), a central nucleotide binding and oligomerization (NACHT) domain, bounded by C-terminal leucine-rich repeats (LRRs) and caspase recruitment (CARD) or pyrin (PYD) domains. In NLRP family, NLRP1 (and its derived CARD8) sensors alone contain a FIIND domain and a CARD domain a C-terminal, NLRP10 sensor alone only consists of a PYD and a NACHT domains, while the remaining are similar, but activated by different stimuli. Numerous NLRP stimuli can trigger NLRP activation to an assembled inflammasome, and these stimuli include microbe-derived signals (commensal bacteria and commensal fungi), pathogen-derived signals (foreign bacteria, fungi, parasites, and viruses), and host-derived signals (ion flux, mitochondrial dysfunction and damages, ROS, and metabolic factors). A *bona fide* assembled NLRP inflammasome consists of a cytosolic a NLRP gene (the sensor), an apoptosis-associated speck-like (ASC) protein containing a caspase activation and recruitment domain, CARD (the adaptor), and a cysteine protease caspase-1 (CASP1) (the effector)

As reviewed by Schroder and Tschopp [3], the core structure of the NLR family consists of a central nucleotide-binding and oligomerization (NACHT) domain, bounded by C-terminal leucine-rich repeats (LRRs) and N-terminal caspase recruitment (CARD) or pyrin (PYD) domains. Based on phylogenetic analyses of NACHT domains from NLR family, it was revealed that the NLR family could be classified into 3 distinct NLR subfamilies: the NODs (NOD1-2, NOD3/ NLRC3, NOD4/NLRC5, NOD5/NLRX1, CIITA), the IPAF, consisting of IPAF (NLRC4) and NAIP, and the NLRP subfamily. The NLRP subfamily is composed of 14 proteins, named NLRP1-14, from which the NLRP-associated inflammasomes have been named, respectively (Fig. 1). In principle, the activation of NLRP inflammasomes initiates the oligomerization of sensor proteins, facilitating the assembly of the inflammasome through homotypic interactions between PYD-PYD or CARD-CARD domains, which in turn recruits and activates pro-inflammatory caspase-1 protease. The activated caspase-1 then triggers pyroptosis by cleaving GSDMD, leading to the release of IL-1β and IL-18. However, because of the complexity of the activation pathway, the activation mechanism for NLRP inflammasome by different activators remains to be addressed [2, 13].

In this review, we have gathered recent knowledge on the role of NLRP inflammasomes in health and diseases, their structural organization, and their mechanism of activation and regulation. Moreover, by describing the etiology of aberrant inflammasome activation that leads to diseases, we highlighted current therapeutic inhibitors and opened future possible strategies that can better help in mitigating the activation of pathogenic inflammations.

## Overview of NLRP inflammasomes: from health to disease

### NLRP inflammasomes in health

In general, NLRP inflammasomes are intracellular hetero-oligomeric proteins that play an essential role in innate immunity, providing a rapid and efficient response against DAMPs released from infected, stressed, dead, and dying cells and PAMPs from bacterial and viral infections. By recognizing these signals of infection or tissue damage (PAMPs and DAMPs), activated inflammasomes induce the production of pro-inflammatory cytokines, which recruit immune cells to the site of infection and injury, trigger inflammation, and promote tissue and organ repair. NLRP inflammasomes consist of 3 multiportion protein complexes (a stimuli sensor protein [NLRP], an adaptor protein [ASC], and an effector protein [pro-caspase-1]) that recognize a plethora of danger signals (PAMPs and DAMPs) by NODs (such as NLR that contains a C-terminal caspase-recruiting domain [CARD]), and modulate caspase-1 activation, which consequently induces pyroptosis for the host health preservation, in theory [19, 20] (Fig. 1). In other words, inflammasome-induced pyroptosis destroys and eliminate infected and damaged cells in order to prevent infection spread and restore or maintain tissue homeostasis.

Mechanistically, upon assembly-based activation, NLRP inflammasomes trigger cleavage of pro-IL‐1β and pro-IL‐18 by activating caspase proteins into IL‐1β and IL‐18, their respective mature forms. Mature IL‐1β binds to its receptor and the interaction triggers leukocyte infiltrations, lymphocyte activation, and acute phase protein induction, and favors a chemotactic environment (secretion of inflammatory factors and chemokines at the inflammatory site), which in turn, promotes inflammatory response against the specific stimuli that have induced inflammasome assembly [2, 21, 22]. Simultaneously, mature and activated IL-18 induces the production of cell stress-associated components, including nitric oxide and reactive oxygen species, which increases chemotactic environment and recruits immune cells. Besides, activated caspase-1 from the NLRP inflammasome complex induces cleavage of gasdermin D (GSDMD) into its activated form, which incorporates into the cell membrane and creates pores via its free N-terminal end, and consequently causes infected cell swelling and inflammatory-related death known as pyroptosis [23].

Constitutively, GSDMD consists of a central short linker region bounded by an N-terminal domain of GSDMD (GSDMD-N_term_) and a C-terminal auto-inhibition domain. Notably, the GSDMD-N_term_ is the active cell death domain. Cleavage of GSDMD by activated caspase-1 at GSDMD C-terminal removes the auto-inhibition domain and releases an activated GSDMD with an available N-terminal, which binds to the inner leaflet cell membrane phosphatidylinositol phosphates and phosphatidylserine. This binding results in GSDMD oligomerization and its insertion within the cell membrane, forming 10 [14] nm pores that cause pyroptosis [24]. Note that GSDMD-caused pyroptosis also favors release of mature IL‐1β and IL-18 via nonconventional secretion, favoring health-associated NLRP inflammasome inflammatory response. The cell debris comprising degraded antigenic peptides is cleared through blood circulation to restore tissue or organ homeostasis and host health. Summarily, through pyroptosis signaling, NLRP inflammasomes contribute to the clearance of pathogens and the maintenance of tissue homeostasis.

To date, there are about a dozen species within the NLRP family, precisely fourteen [25]. NLRP1 (along with its derivative CARD8) sensors possess exclusively a FIIND domain and a CARD domain at the C-terminal, whereas the NLRP10 sensor alone consists only of PYD and NACHT domains. The remaining members share similar structural domains, including an N-terminal PYD, a middle NACHT domain, and a C-terminal LRR region (Fig. 1). A variety of stimuli can trigger NLRP activation and lead to the formation of an assembled inflammasome. NLRP1 and NLRP3 inflammasomes are the main extensively studied and well-described so far, whereas other NLRPs including NLRP2, NLRP6, NLRP7, NLRP9, NLRP10, and NLRP12 have also been identified to form inflammasomes, and because of their important roles in health and diseases as well, they are in the middle of scientific investigations.

### NLRP inflammasomes in disease

**Fig. 2:**
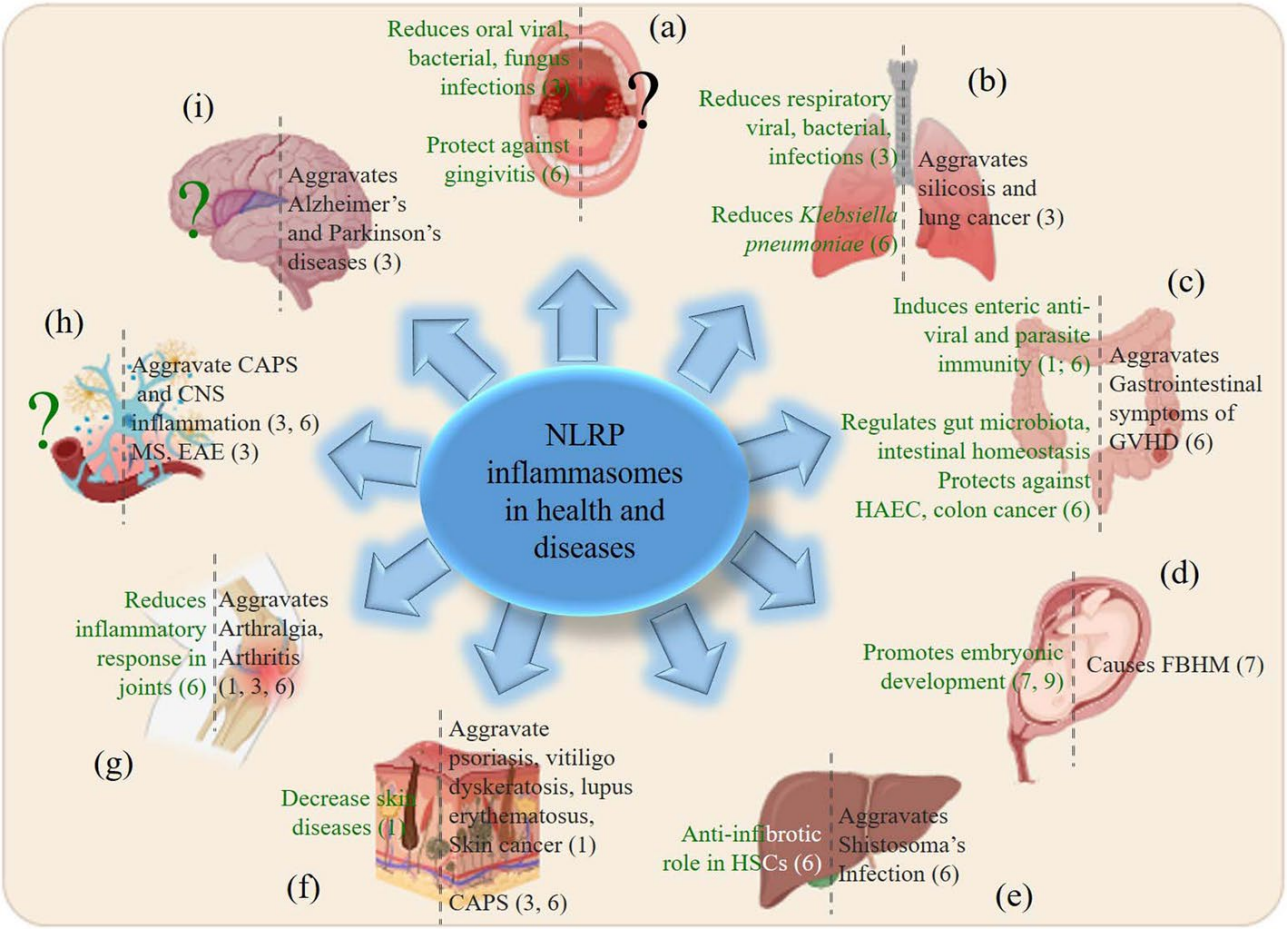
Roles of NLRP in health and diseases in different organs and tissues. Schematic representation of the different functions of NLRP in (**a**) oral cavity, (**b**) lungs, (**c**) digestive system, (**d**) pregnancy and fetus, (**e**) liver, (**f**) skin, (**g**) joints, (**h**) peripheral and central nervous system, (**i**) brain. Functions shown in green represent the NLRP inflammasome-dependent protective response (or NLRP beneficial roles in health) and functions shown in black represent the NLRP inflammasomes-associated diseases (or NLRP detrimental roles in diseases). Numbers in brackets indicate the different NLRPs associated. “?” indicates unknown roles of NLRP inflammasomes

Despite the benefic role played by NLRP inflammasomes to clear pathogens (or foreign bodies) and restore cell and organ health and homeostasis, NLRP inflammasomes are the main leaders of chronic diseases and health state degradation by chronic inflammation. So, the limit between beneficial and pathogenic inflammations is very thin (Fig. 2). Indeed, aberrant activation and dysfunction of NLRP inflammasomes are health-threatening and responsible for chronic inflammation. Chronic inflammation is characterized by a steadily high or an increasing production of pro-inflammatory cytokines (yielding cytokine storm), persisting for a long time beyond the infection clearance (or in absence of infections) and where the immune response continues to pump out white cells and release chemical messengers. The chronic inflammatory response is generally and clinically characterized by death of cells at the infection site. It is noteworthy that chronic inflammation, caused by NLRP inflammasomes, is involved in organ damage, persistence of pre-acquired diseases, and the disease development process of several conditions, which include but are not limited to Alzheimer’s disease, asthma, cancers, heart diseases, rheumatoid arthritis, ankylosing spondylitis, and diabetes, and known as chronic diseases [26]. Most NLRP inflammasome dysfunctions are caused by mutations within one gene regulating the formation of NLRP inflammasome and cause rare disease conditions known as cryopyrin-associated periodic syndromes (CAPS) [27]. Commonly known as gain-of-function mutations, these gene modifications usually occur in the NLRP proteins, and induce a dysfunction or dysregulation of the NLRP inflammasomes.

Note that mutations in other NLRP inflammasome components have not been observed so far, which suggest that aberrant inflammasome activation nondependent to NLRP mutations, may be caused by health condition-related inflammasome dysregulation. For instance, a recent study demonstrated that severe COVID-19 patients are characterized by an immune response heterogeneity, also known as a reduced immune system fitness [28]. This condition is attributed to the inability of the immune system (challenged by the virus) to properly downregulate the NLRP3 inflammasome activation. Thus, unhealthy people with reduced immune response fitness display a dysregulated NLRP3 inflammasome activity, characterized by a steady and persistent inflammatory response, worsening the infection [28]. Moreover, another study showed that type 2 diabetics with chronic glycemic dysregulation demonstrate a dysregulated NLRP inflammasome, characterized by a prolonged upregulated inflammasome activation, causing diabetic retinopathy [29].

At the molecular level, in already activated inflammasome state (by either aforementioned etiology), persistence inflammatory response is usually caused by accumulation of a variety of NLRP inflammasome danger signals in threatened organs and tissues and leads to systemic chronic inflammation and chronic diseases [26]. Even though molecular causes exacerbating danger signal persistence are not well described, it is thought, as aforementioned that many biological, social, and environmental factors are involved in inflammation persistence or resurgence of acute inflammation [28, 29] that disrupts organ or tissue normal physiology and breakdown immune tolerance, which leads to chronic inflammatory diseases. Therefore, being linked to NLRP inflammasome activation, it is of crucial need to deeply understand the mechanisms of activation for each NLRP inflammasome, as they would disclose regulation check points or target molecules for therapeutics development.

## NLRP1 inflammasome

### NLRP1 inflammasomes and its role in health and innate immunity

NLRP1 was the first member of the NLRP family to be identified and shown to form an inflammasome complex and induce activation of caspase-1. NLRP1 inflammasome was first described in human microglia and neuronal cells (where NLRP1 expresses the most) before they were characterized in mice [5, 30], in which three, but four paralogs of NLRP1 (NLRP1*a*-*d*) are found so far [25, 31–34]. These mouse NLRP1 paralogs display different functionalities: in some mouse strains, such as the 129S mouse strains, only NLRP1*b* is functional, while the ability of the rest to stimulate the inflammasome pathway is unknown, therefore those are pseudogenes [35]. Interestingly, it was found that human and mouse NLRP1s are quite divergent in their protein domain structure, which has rendered difficult and hampered the functional studies and analyses of NLPR1s [36]. Specific studies were conducted in both mice and human cells to explore the specific and typical functions of NLRP1s in each organism. The results from mouse studies revealed that mouse and human NLRP1 display distinct and overlapping functions, which could only partially contribute to disclosing the role of human NLRP1. Researchers keep studying NLRP1s to understand their specific functionalities in human cells and their roles in health and diseases.

While the NLRP1 inflammasome and its direct effect on health were poorly understood until recently compared to that of the NLRP3 inflammasome, many studies have started to deeply investigate and disclose its role in the innate immune response in mice and humans. A recent study [37] investigating the activation mechanism of inflammasomes in intestinal epithelial cells (IECs) infected with transmissible gastroenteritis virus (TGEV) showed increased levels of pro-inflammatory cytokines (IL-1β, and IL-18) in both IECs and TGEV-infected tissues, with increased transcription and expression of Nlrp1 gene and NLRP1 protein, respectively, and an upgraded activation of caspase-1. Additionally, the TGEV infection-associated high activation of NLRP1 also acts as an interferon-stimulated gene to counteract enterovirus TGEV infection [37]. In other viral infections, such as in *Picornaviridae* family-related infections and double-stranded RNA Semliki Forest virus infections, NLRP1 has been identified as a sensor that triggers and regulates the protective innate immune response [38, 39].

The role of NLRP1 inflammasome in health and diseases has been investigated. NLRP1 inflammasome has been associated with numerous disease releases. Specifically, certain NLRP1 variants including NLRP1 rs12150220 polymorphism, found in skin inflammatory diseases, such as vitiligo-associated autoimmune diseases, like Addison’s disease, type 1 diabetes, and systemic lupus erythematous, have been associated with a decreased occurrence risk of these diseases [40]. There are many other studies that have reported the involvement of NLRP1 inflammasome in diverse infection-associated immune responses (Fig. 2). How the NLRP1 inflammasome is activated still remains a subject of debating hypotheses. Nevertheless, as we describe bellow, recent reports have raised crucial and concluding facts about the mechanism of NLRP1 inflammasome activation.

### Structure of NLRP1 inflammasome

**Fig. 3:**
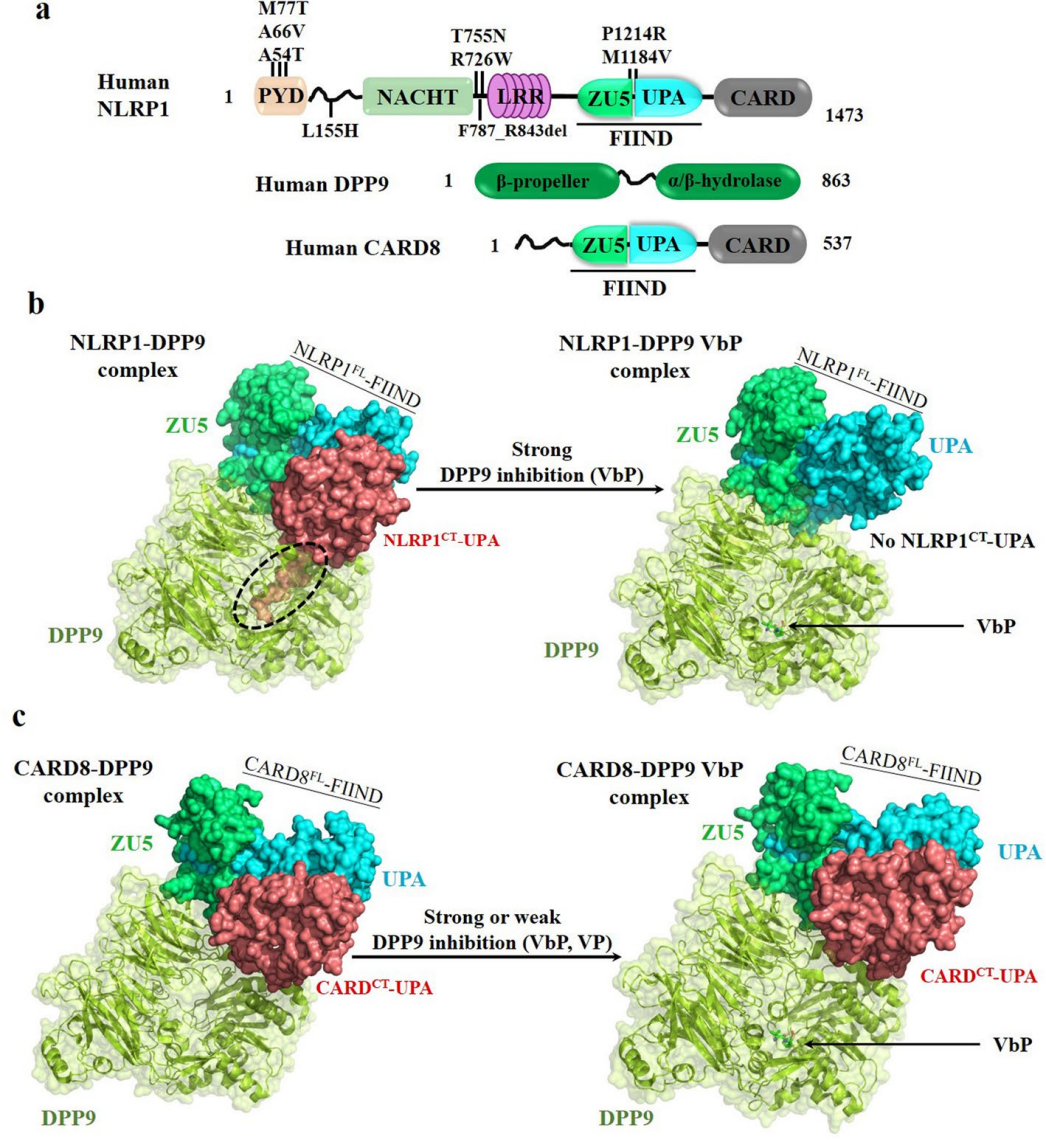
Structural features of human NLRP1. **a** Domain organization of NLRP1, DPP9 and CARD8. Human NLRP1 and CARD8 autoproteolysis sites are shown between the ZU5 and UPA subdomains of the FIIND. The dotted black circle shows the N-terminus of NLRP1CT-UPA folded into the DPP9 active-site tunnel. **b** Cryo-EM structure of the hNLRP1^FL^-DPP9-hNLRP1^CT^ ternary complex. NLRP1 directly bonds to the DPP9 active site and strong DPP9 inhibitors (e.g., VbP) are required to displace the inserted peptide of hNLRP1^CT^ in the DPP9 catalytic pocket and destabilize the ternary complex. **c** Cryo-EM structure of the hCARD8^FL^-DPP9-hCARD8^CT^ ternary complex. Unlike human NLRP1, the precisely mechanism of the ternary complex destabilized by DPP9 inhibitors (e.g., VbP, VP) remains unknown because CARD8 does not directly bond to the DPP9 active site

As a member of the NLR family, NLRP1 in humans is the largest member. The structural architecture of human NLRP1 is unique. It comprises a pyrin domain (PYD), followed by a nucleotide-binding domain (NBD), five tandem LRR domains, a ‘function to find’ (FIIND) domain, and a carboxy-terminal caspase activation and recruitment domain (CARD) [41] (Figs. 1 and 3). The PYD and CARD domains in human NLRP1 belong to the death domain (DD) superfamily for interactions among sensors, adaptors, and caspase-1 [42]. Although human NLRP1 simultaneously contains both PYD and CARD domains, the C-terminal CARD, but not the auto-inhibitory PYD, is a protein–protein interaction domain for inflammatory signal transduction [43]. The interaction between the CARD domain of NLRP1 and adaptor protein ASC is necessary to facilitate NLRP1 inflammasome assembly and activation [44]. Additionally, the FIIND domain is encoded by only 3 proteins, including NLRP1 [45], the CARD-containing protein 8 (CARD8) [46], a p53-induced protein with a death domain [47], and not found in any other NLR in the human proteome. The spontaneous proteolytic cleavage of FIIND between ZU5 (found in ZO-1 and UNC5) and UPA (conserved in UNC5, PIDD, and ankyrins) subdomain generates a large N-terminal fragment and a smaller C-terminal fragment, which remain associated by non-covalent interactions [48]. The self-cleavage between phenylalanine 1212 and serine 1213 within the FIIND domain is required to activate human NLRP1 [45, 49]. Furthermore, studies have shown that the large linker region between PYD and NOD is critical for proteolytic cleavage or post-translational modification of NLRP1 [50–52].

The human genome contains seven different isoforms of NLRP1 produced by alternative splicing, each with protein sequence alterations that are located in the C-terminal region. Several mammalian species (e.g., rodents and primates) express extensively diversified NLRP1 protein among different species. Unlike human NLRP1, the domain organization of three murine orthologues NLRP1*a*, NLRP1*b*, and NLRP1*c*, all lack an N-terminal PYD. Although bioinformatics analyses revealed that NLRP1C is a pseudogene [33], mouse NLRP1A and NLRP1B can directly recruit the effector inflammatory molecule proCaspase-1 independent of ASC [53]. Polymorphisms of mouse NLRP1B have been identified, with five different Nlrpb alleles present in inbred mouse strains [31]. In addition to the Nlrpb allele, the Nlprld-f alleles were recently described with little knowledge of their functionality [34]. To our knowledge, the differences in NLRP1 alleles result in a genetic diversification of NLRP1.

### Molecular mechanism of NLRP1 inflammasome activation

**Fig. 4:**
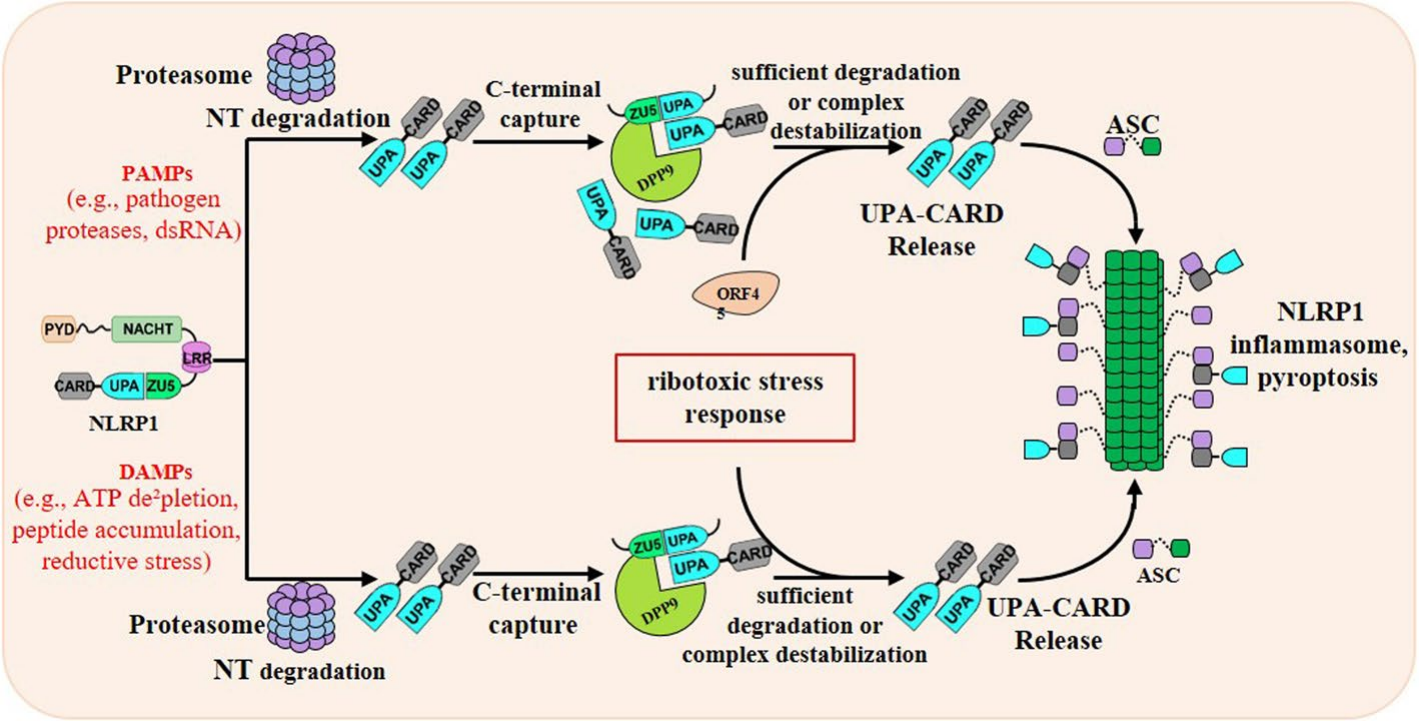
The mechanisms of human NLRP1 inflammasome activation by PAMPs and DAMPs. The proteasome-mediated degradation of N-terminus of NLRP1 liberates the C-terminal UPA-CARD from autoinhibition. The acceleration of degradation of NLRP1 by several PAMPs (for example, pathogen proteases) overwhelms the DPP9 ternary complex checkpoint to oligomerize into an inflammasome. On the other hand, the destabilization of the DPP9 ternary complex by several DAMPs (for example, peptide accumulation) releases the C-terminal functional domain of NLRP1 to assemble into an inflammasome. There are two distinct signals-sufficient degradation and repressive complex destabilization-create an unstable state to active NLRP1 inflammasome

NLRP1 inflammasome was the first described intracellular canonical inflammasome that could activate inflammatory caspase-1 [5], yet its activation pathway remained poorly understood for a long time. Recently, NLRP1 inflammasome has increasingly become the focus of innate immune recognition pathways, and our understanding of the modulators and regulators of NLRP1 inflammasome assembly and function has advanced remarkably [54]. Recent emerging studies have started to shed light on the natural physiological stimuli and biological purpose of NLRP1 inflammasome, for a better understanding of the activation pathway and its role in health and disease. Many researches have highlighted the importance of NLRP1 inflammasome in the pathogenesis of various inflammatory pathologies. Moreover, numerous auto-inflammatory diseases connect with increases in NLRP1 inflammasome activity due to dysregulation of NLRP1, including vitiligo, systemic sclerosis, melanoma and Addison’s disease [45, 55] (Fig. 2). Novel insights on the mechanisms between NLRP1 inflammasomes and auto-inflammatory diseases at the molecular level are discussed hereinafter (Fig. 4).

NLRP1 undergoes post-translational auto-processing within the FIIND domain to generate two non-covalently associated polypeptides sequestered by dipeptidyl peptidase DPP8 and DPP9 (DPP8/9). Under the stimulation of activation signaling of NLRP1, such as the disruption of NLRP1 auto-inhibitory state by Val-boroPro (VbP), the NLRP1 exposes a destabilized N-terminus of NLRP1 that can be recognized by the UBR2/UBE2O-mediated degradation machinery. Subsequent proteasome-mediated degradation of the N-terminal protein fragment removes the self-inhibition function of NLRP1 and liberates the C-terminal UPA-CARD, which is important to recruit adapter protein ASC. The UPA-CARD co-assembles with ASC via homotypic CARD-CARD interaction to form higher-order signalosomes for pro-inflammatory caspase-1 protease recruitment and activation [44]. Dimerized or activated caspase-1 subsequently induces pyroptotic cell death through proteolytic activation of GSDMD and releases inflammatory cytokines, such as IL-1β and IL-18 [56].

NLRP1 inflammasome is a death-fold containing inflammasome, and its PYD and CARD domains are members of the death domain superfamily. The death domains frequently mediate the oligomerization to form a filamentous platform triggering activation of inflammatory caspase-1 in inflammasome. The filamentous structures of the PYD domain of ASC, the CARD domain of ASC and caspase-1 reveal insights into the DD-mediated assembly mechanism [56–59]. The three asymmetric interaction types of the DD superfamily (Type I, II and III), which have distinct interaction interfaces in each DD superfamily member, are required to assemble complex macromolecular structures. Significantly, we demonstrated that NLRP1^CARD^ co-folds with ASC^CARD^ by these three conserved interfaces and proposed a “Mosaic model” to explain the danger signal transduction amplification in NLRP1 inflammasome [44]. Recent studies also reported the polymerization and assembly mechanism of the C-terminal functional domain of NLRP1 and its analogous CARD8 [60, 61]. In the UPA-CARD filament structure of NLRP1, the CARD domain is located in the core of the filament, and the UPA subdomain, located outside the core filament, is disordered because of a long flexible linker between UPA and CARD. The UPA subdomain enhances the polymerization of the CARD domain, facilitating the filamentous complex formation. Another NLRP1 CARD filament structure comprises CARD dimers outside the core CARD filament that differs from other CARD filaments [60, 61]. These higher-order filamentous complexes have deep biological implications in inflammasome activation and signal transduction amplification.

#### Physiological activation of NLRP1 inflammasome by PAMPs

Lately, murine-based studies provided more decisive insight into the activation mechanism of NLRP1 (Fig. 4). The Lethal Factor (LF) from *Bacillus anthracis* of PAMPs is the best-characterized NLRP1 activator that activates a subset of murine NLRP1B (mNLRP1B) and rat NLRP1 (rNLRP1B) proteins [62, 63]. Studies on the mNLRP1B inflammasome along with the identification of the natural physiological stimuli LF of *Bacillus anthracis* represent a major breakthrough in our understanding of NLRP1 inflammasome activation mechanism [31]. The specific activator anthrax lethal toxin consists of a protective antigen and a lethal factor that could trigger NLRP1B inflammasome assembly to activate caspase-1 and secret IL-1β, but not human NLRP1 inflammasome. In this regard, the FIIND domain of mNLRP1B first auto-proteolysis to produce two non-covalently associated N-terminal and C-terminal fragments. LF mediates the cleavage of the mNLRP1B amino-terminal domain to expose a destabilizing neo-N terminus [64, 65]. Subsequent N-end rule E3 ligase UBR2 recognizes and ubiquitinates the destabilizing N-degron, which is rapidly degraded by proteasome-mediated degradation machinery [66]. The active C-terminal fragment is not degraded because of the break within the FIIND domain but rather is liberated to recruit the pro-inflammatory caspase-1 protease. Activated caspase-1 subsequently induces pyroptotic cell death and inflammatory cytokine releases, such as IL-1β and IL-18 [67]. The unifying mechanism of proteasome-dependent NLRP1 inflammasome activation has been named “functional degradation” [50, 67]. Recently, Sandstrom and co-workers found that IpaH7.8 of *Shigella flexneri* leads to NLRP1B-dependent IL-1β production in the reconstituted NLRP1B inflammasome system [67]. In mice, *Shigella* infection induces cytokine production and causes macrophage pyroptosis by IpaH7.8-mediated NLRP1B activation. These data are consistent with the “functional degradation” model of NLRP1 inflammasome activation and suggest that the N-terminal fragment of NLRP1 might act as a kind of tripwire in the detection of pathogens. Specifically, the N-terminus of NLRP1 might serve as a decoy and is sensed by pathogenic protease activity that could destroy mammalian NLR receptors to evade detection by the innate immune system. But the degradation of the N-terminus activates NLRP1 inflammasome and causes an effector-triggered immunity to achieve the function of efficient detection and clearance for foreign pathogens.

The activation trigger and activation mechanisms of human NLRP1 and mouse NLRP1 are highly divergent. The molecular activation mechanism of human NLRP1 remains enigmatic because no physiological activators of human NLRP1 have been found for a long time. Recent studies have started to describe its danger activation signals and illuminate its functional relevance. First, it has been shown that an enteroviral 3C protease of human rhinovirus (HRV) was identified as a physiological activator of human NLRP1 [38, 68]. The authors discovered that 3C protease directly cleaves the human NLRP1 between Q130 and G131 in the N-terminal fragment. 3C protease cleavage of NLRP1 leads to the degradation of N-terminal fragment and liberation of C-terminal UPA-CARD. As mentioned above, the C-terminal functional domain will result in the assembly of active inflammasome and subsequent cytokine release, which is in line with the “functional degradation” model. The 3C protease was identified as the first pathogen-associated activator of human NLRP1 in human primary airway epithelial cells. Second, a recent manuscript reported that SARS-CoV-2 3CL protease NSP5 could trigger an NLRP1-mediated inflammasome response to decrease the production of infectious viral particles [69]. Mechanistically, human NLRP1 is cleaved at the Q333 site by SARS-CoV-2 3CL protease and activated by the “functional degradation” model, similar to what has been found for enteroviral 3C protease. Notably, SARS-CoV-2 3CL protease NSP5 acts as a virulence factor against GSDMD-dependent pyroptosis, but it promotes cell death by caspase-3/GSDME-mediated pyroptosis pathway upon SARS-CoV-2 infection. Third, the other extraordinarily different activator, long double-stranded RNA (dsRNA), is discovered to activate NLRP1 inflammasome in keratinocytes [39]. Semliki Forrest virus (SFV), a positive-sense single-stranded RNA virus, was found by testing different types of viruses. The autoproteolytic and proteasome activity were necessary for dsRNA-induced human NLRP1 activation, suggesting the N-terminal “functional” proteasomal degradation is involved in this process. Using recombinant proteins, human NLRP1, but not murine NLRP1B could directly interact with dsRNA by its NACHT-LRR domains with high affinity. DsRNA binding to NLRP1 enhanced ATPase activity to achieve a conformational switch that releases an active carboxy-terminal fragment. Fourth, Yang and co-workers described a proteasome-independent activation mechanism of NLRP1 inflammasome in 2022 [70]. The tegument protein ORF45 of Kaposi sarcoma-associated herpesvirus (KSHV) induced NLRP1-mediated inflammasome activation in human epithelial and macrophage-like cells. Mechanistically, there are two different NLRP1 auto-inhibitory complexes, the N-terminus of NLRP1 (hNLRP1^NT^)- C-terminus of NLRP1 (hNLRP1^CT^) and hNLRP1^FL^-DPP9-hNLRP1^CT^, respectively. The interaction between the Linker1 region (amino acids 93–327 in human NLRP1) and the UPA subdomain in NLRP1 is critical for the two auto-inhibitory complexes in cells. KSHV ORF45 unlocked the two auto-inhibitory complexes by disrupting the Linker1-UPA interaction, which promoted the hNLRP1^CT^ inflammasome assembly. The NLRP1 activation process was conserved in primates but not in rodents. In addition, mouse NLRP1B also responds to *Toxoplasma gondii* to activate NLRP1B inflammasome, which protects against parasite infection in mice [71–73]. In recent studies, it was revealed that bone marrow macrophages undergo lytic cell death and IL-1β secretion during *T. gondii* infections. The mechanism of NLRP1B activation by *T. gondii* infections may not correlate with responses to lethal anthrax toxin and needs further studies with the development of new technology. In conclusion, these results further highlight the emerging functions of NLRP1 inflammasome in host defense.

#### Physiological activation of NLRP1 inflammasome by DAMPs

Because NLRP1 recognizes various modalities through the different ligand-binding domains and the different molecular modes of action, the activation mechanisms of NLRP1 inflammasome are extremely complex. In addition to sensing PAMPs, NLRP1 inflammasome has been reported to recognize DAPMs in recent years (Fig. 4). Specifically, mouse NLRP1B is known to lead to excessive IL-1β secretion and pyroptosis of cells by cellular perturbations, such as ATP depletion [74]. The NBD domain of NLRP1B with a nucleoside triphosphatase activity allows ATP binding to detect cellular ATP deprivation, and mutations in the Walker A site of NLRP1B cause the spontaneous NLRP1B inflammasome activity. It should be noted that the activation of NLRP1B inflammasome by glycolysis and oxidative phosphorylation inhibitors is consistent with the “functional degradation” model because IL-1β production could be blocked by proteasome inhibition. Nevertheless, the detailed ATP-dependent NLRP1B activation mechanism remains unknown.

In addition, Elizabeth and colleagues identified that the specific cellular danger signal, cytosolic peptide accumulation, could be detected by NLRP1 and CARD8 inflammasome [75]. They found that several different and well-characterized agents interfere with protein folding and accelerate the N-terminal fragment of NLRP1 degradation. The production of peptides with N-terminal XP sequences (X stands for any amino acid) destabilizes the NLRP1-DPP9 ternary complexes to trigger inflammasome activation, like what has been found by VbP. Notably, these proteotoxic drugs, such as MeBs (Bestatin methyl ester), BFA (brefeldin A) and GA (geldanamycin), only stimulate the degradation of the N-terminus of NLRP1, but they could not activate the NLRP1 inflammasome due to the inhibition by NLRP1-DPP9 ternary complexes.

On the other hand, these agents synergize with VbP to trigger inflammasome activation both upstream and downstream of the proteasome. Interestingly, recent research showed that the ATPase activity of the NLRP1 NACHT domain and the interaction between the NACHT-LRR domain of NLRP1 and oxidized thioredoxin-1 (TRX-1) are required to restrain the NLRP1 inflammasome activation [76]. Negative regulation of NLRP1 inflammasome by oxidized form of TRX1, but not reduced TRX1, suggests that reductive stress might act as a danger signal to trigger NLRP1 inflammasome activation. These same investigators later identified a panel of related radical-trapping antioxidants that could accelerate the proteasome-dependent degradation of auto-inhibition domains of NLRP1 and CARD8 by inducing reductive stress, similar to what has been found for peptide accumulation [77]. Reasonably, reductive stress and peptide accumulation could increase the activation level of NLRP1-mediated inflammasome more than either signal alone.

Furthermore, these radical-trapping antioxidants synergize with VbP, thus initiating more inflammatory cytokine secretion and pyroptotic cell death. Because these radical-trapping antioxidants also prevent an iron-dependent form of cell death named ferroptosis, a subsequent study reported that ferroptosis is linked with NLRP1 inflammasome in a model of oxidative stress [78]. The results of cytological experiments showed that the extent of NLRP1 inflammasome is reduced or increased with changes in ferroptosis activity, and the interactive relationship of NLRP1 inflammasome and ferroptosis is demonstrated under oxidative stress. Consistent with this mechanism above, O_3_, one of the most toxic pollutants, could be sensed by NLRP1 inflammasome in human keratinocytes [79]. Oxidative stress event caused by O_3_ exposure induces UBR2-mediated ubiquitination and proteasomal degradation of NLRP1, resulting in NLRP1 inflammasome assemble and inflammatory cytokines release.

Finally, the constitutive expression of NLRP1 in keratinocytes perhaps indicates that NLRP1 is engaged in response to ultraviolet B (UVB) exposure [80, 81]. UVB and toxin-induced ribotoxic stress response (RSR) were recently discovered to induce human NLRP1 inflammasome activation by direct phosphorylation [82, 83]. Mechanistically, low-irradiance UVB results in the activation of RSR kinase ZAKα and its downstream effector p38 to directly phosphorylate the disordered linker region between PYD and NACHT domains in human NLRP1. Hyperphosphorylation of disordered region by ZAKα and p38 induce inflammasome assembly and NLRP1-driven pyroptosis. Importantly, they found that stimulation of ZAKα and p38 is sufficient to induce NLRP1 activation in a DPP8/9-independent manner. They also found that NLRP1 is a versatile receptor that could integrate diverse stress signals by its different regions. Nevertheless, these findings suggest that NLRP1 is a complex receptor that responds to pathogens infection but also engages in maintaining host homeostasis.

#### DPP8/9 inhibitors: the common activator for human and mouse NLRP1 inflammasome

VbP is a small molecule inhibitor of fibroblast activating protein (FAP) and dipeptidyl peptidase family, including DPP4, DPP7, DPP8 and DPP9. VbP is originally used to induce cytokine production and stimulate anti-cancer immune responses in mice [84, 85]. In 2018, VbP was found to induce NLRP1B-mediated pyroptotic cell death in mouse macrophages [86]. Consistent with the conserved nature of DPP8/9, Zhong et al*.* discovered that human NLRP1 mediates VbP-induced inflammasome activation in keratinocytes [87]. Since this report, VbP has been shown to activate functional rodent alleles and human NLRP1, thus becoming the first known universal NLRP1 activator.

Subsequent studies investigated the VbP-induced activation mechanism of NLRP1 inflammasome. DPP9 was demonstrated to bind to NLRP1 under steady-state conditions using immunoprecipitation assays, in which the interaction was abolished in VbP-treated cells [87]. The interaction of DPP9 with hNLRP1 and mNLRP1 is common, as the DPP9-binding domains in NLRP1 are the most homologous FIIND domains. For the inhibitory effect on NLRP1 inflammasome, the binding of DPP9 and its catalytic activity are a prerequisite. Recently, two back-to-back articles report cryo-EM structures of NLRP1-DPP9 complex, respectively [88, 89]. Interestingly, the NLRP1-DPP9 complex is a ternary complex that consists of DPP9, full-length NLRP1 and the UPA-CARD of NLRP1. In the tripartite complex, the FIIND and UPA subdomain of NLRP1 are visible in the cryo-EM map density, while other domains are not discernible because of the flexible linker. For the first FIIND domain of full-length NLRP1, this structure revealed that the first β-strand of the UPA subdomain inserts into the ZU5 fold, like the autoinhibited FIIND domain. Surprisingly, DPP9 formed a homodimer that captures the second UPA-CARD of NLRP1 to suppress UPA-CARD from self-oligomerization during homeostatic protein turnover. For the second NLRP1 molecule, only the UPA subdomain was discernible, and the disordered N-terminal region (S1212-N1224) folds into the DPP9’s active-site tunnel, similar to the substrate-bound DPP9 structure (Fig. 3b). However, the N-terminus was not cleaved by recombinant DPP9 due to the difference in the binding pose with how substrates bind. Therefore, the results suggest that NLRP1 could be sequestered by DPP9 rather than act as a bona-fide substrate.

Next, the structure of NLRP1-DPP9 in the presence of VbP was solved to reveal that VbP forms a covalent bond with catalytic S730 residue of DPP9 and displaces the interaction between DPP9 and UPA of NLRP1 from the substrate tunnel, which is consistent with the reports that VbP diminishes the interaction of NLRP1 and DPP9 [87]. Another notable aspect of this complex is that the UPA-UPA interaction of two NLRP1 molecules is important in both NLRP1 repression and NLRP1 activation. Mutations on the interface led to the autoactivation of NLRP1 and abolished the UPA-mediated oligomerization, which aligns with UPA-mediated CARD filament formation [60, 61]. Altogether, these data provide important insights into how DPP8/9 negatively regulates NLRP1 inflammasome activation.

### NLRP1 inflammasome regulation and dysfunction

#### Regulation of NLRP1 inflammasome activation

As previously described, activation of NLRP1 inflammasome is associated with host immune response against infections. In contrary, inappropriate and/or excessive activation of NLRP1 inflammasome are associated with severe pathologies. Thus, NLRP1 inflammasome activation should be regulated to prevent such pathologies.

The first downregulation of NLRP1-inflammasome activation occurs at the NLRP1 CARD and FIIND domains. In fact, as previously described, to activate NLRP1-inflammasome, human NLRP1 or murine NLRP1b undergo an auto-proteolysis within the FIIND domain, releasing N- and C-terminal fragments, remaining in an auto-inhibited state, where they are ready to recruit NLRP1 cognate to process the activation of NLRP1 inflammasome, IL-1β release, and macrophage pyroptosis. Abolishing FIIND autolytic proteolysis processing activity blocks and downregulates NLRP1 inflammasome activation [45, 90].

Furthermore, in resting macrophages, as well as after infection clearance, NLRP1 inflammasome should be shut down, and its activation, downregulated. Bcl-2 and Bcl-X_L_ proteins, initially known to regulate apoptosis, were found to interact with NLRP1 protein (but not with other NLRP proteins) and prevent activation of NLRP1 inflammasome [48]. Specifically, Bcl-2 and Bcl-X_L_ proteins would form a complex (Bcl-2/X_L_) that recognizes and binds to NLRP1, which suppresses the NLRP1-mediated activation of caspase-1 and subsequently prevents production of IL-1β [91]. The negative regulation of Bcl-2 and Bcl-X_L_ was also demonstrated in immune escape mechanisms by certain viruses (including Vaccina virus) that produce viral proteins (such as F1L protein) structurally similar to BcL-2 and BcL-X_L_, which interact with NLRP1 and downregulate NLRP1 inflammasome activation, which is however favorable for viral replication and spread [48, 92]. Specifically, like BcL2/XL protein complex, F1L protein of Vaccina virus binds to the ATP-binding site on NLRP1, impeding ATP recruitment by NLRP1 and blocking activation of NRLP1 inflammasome. In vitro assay with Vaccinia virus mutants that lack F1L demonstrates a significant production of IL-1β production in human THP-1 cells, which confirms the negative NLRP1 inflammasome regulation [92]. Besides that, the intracellular ORF63 protein was also found to downregulate NLPR1 inflammasome activation and impede production of IL-1β, through binding with NLRP1 and NLRP3, in THP-1 macrophages that were sensed and stimulated by MDP (muramyl dipeptide). In presence of a cognate NLRP1 inflammasome stimulus, downregulation by Bcl-2/X_L_ complex protein and ORF63 is by-passed to prevent their binding to NLRP1 and allow activation of NLRP1 inflammasome [48]. However, the mechanism of blocking the homeostatic effect of Bcl-2, Bcl-X_L_, and ORF63 remains unclear.

#### NLRP1 inflammasome dysregulation and associated diseases

Indeed, when the regulation of NLRP1 inflammasome activation is disrupted, it can lead to the occurrence of disease. Many studies have found that NLRP1 can produce pro-inflammatory cytokines after activation, mediate inappropriate inflammation and participate in a variety of physiological immune responses, suggesting that NLRP1 inflammasome contributes in many disease development processes (Fig. 2 and Table 1). Specifically, this aberrant activation of NLRP1 inflammasome have been associated with mutations found in NLRP1, which in turn have mainly been associated with occurrence of diseases, including severe chronic obstructive pulmonary disease (COPD) [93], systemic lupus erythematosus [94], type 1 diabetes [95], vitiligo-associated autoimmune diseases [20, 55, 96, 97], inflammatory bowel disease [98], arthritis, dyskeratosis, psoriasis, multiple self-healing palmoplantar carcinomas (MSPCs) and familial keratosis lichenoides chronica (FKLC) [43, 99] (Table 1).

**Table 1:** NLRP inflammasomes-associated diseases. A non-exhaustive list of human diseases associated with NLRP inflammasome dysfunctions. Most, but not all are caused by mutations within inflammasome forming proteins, mainly NLRPs

Diseases	Associated NLRP (Disease characteristics)	Mechanisms of induction	Ref.
COPD, MSPCs, Type 1 diabetes	**NLRP1** (progressive decline in lung function due to airflow limitation [COPD], multiple KAs [MSPCs],	Gain-of-function mutations in NLRP1 gene disrupt auto-inhibition and aberrantly activate NLRP3	[43, 93–95, 99, 100]
SLE	**NLRP1** and **NLRP3** (myriad of immune aberrations from disruption of immunity)	Mutations in NLRP1 and NLRP3 dysregulate NLRP1 and NLRP3 inflammasome	[43, 93–95, 99, 101]
Type 1 diabetes	**NLRP1** (destruction of insulin-producing pancreatic β cells)	Polymorphism in NLRP1 gene (rs11651270 and rs2670660)	[102, 103]
Vitiligo, Dyskeratosis, Psoriasis	**NLRP1** (skin-related inflammatory pathologies and JRRP)	Gain-of-function mutations in PYD (A54T, A66V and M77T) and LRR (F787-R843, T755N) of NLRP1	[55, 96, 97, 104]
FCAS MWS	**NLRP3** (Fever, neutrophilia, multi-organ, inflammation.)	Gain-of-function mutations in NACHT to disrupt auto-inhibition of NLRP3	[27, 105]
Influenza A virus	**NLRP3** (Hyper-inflammation)	Interaction with Viral PAMPs and DAMPs, Influenza A virus M2 protein	[27, 105]
COVID-19	**NLRP3** (Airway and lung inflammation in COVID-19 patients)	Interaction with Viral PAMPs and DAMPs	[27, 105]
Obesity, type 2 diabetes	**NLRP3** (Airway and lung inflammation in COVID-19 patients)	Increased Ceramide, fatty acids	[27, 105]
Asthma	**NLRP3** (Allergic airway inflammation)	Allergic immune signals, nonallergic stimulants	[27, 105]
Irritable bowel disease	**NLRP1, NLRP3, NLRP6, and NLRP7** (Inflammatory DAMPs)	Dysregulation of NLRP1, NLRP3, NLRP6, and Polymorphism in NLRP7 29,211,899	[27, 43, 98, 99]
Nonalcoholic fatty liver disease	**NLRP3** (Choline deficient amino acid, defined- or methionine/ choline-deficient diet-induced nonalcoholic fatty liver disease in mice)	Increased fatty acid, hypercholesterolemia	[27, 105]
Rheumatoid arthritis	**NLRP1 and NLRP3** (Erosive polyarthritis)	DAMPs activation-associated inflammatory	[27, 105]
MS, EAE	**NLRP3** (Autoimmune encephalomyelitis, CNS-associated disorders: physical, mental, and psychiatric challenges)	DAMPs activation-associated inflammatory	[12, 27, 105]
Gout	**NLRP3** (Gouty arthritis. Urate or CPPD crystal-induced inflammation)	Induced by MSU or CPPD crystal Phagocytosis and lysosomal disruption	[27, 105]
Atherosclerosis	**NLRP3** (low-level inflammation; progressive narrow arterial vessels due to cholesterol crystals and white blood cell arterial accumulation)	Induced by cholesterol crystal, Phagocytosis and lysosomal disruption	[27, 105]
Alzheimer’s disease	**NLRP3** (Senile plaques in brain neurons, cerebral neuroinflammation)	Induced by Aβ fibril released by dead neurons, phagocytosed by microglia	[27, 105]
Parkinson’s disease	**NLRP3** (Loss of dopaminergic neurons)	Induced by αSyn fibril released by dead neurons, phagocytosed by microglia	[27, 105]
Silicosis, lung cancer	**NLRP3** (Acute and chronic inflammation, interstitial fibrosis, granuloma formation, and cancer in the lung)	Increased crystalline silica, Phagocytosis. Lysosomal, disruption. Increased ROS	[27, 105]
Tumor, metastasis	**NLRP3** (MCA-induced lung cancer, metastasis)		[27, 105, 106]
FMF	**NLRP3 and NLRP6** (auto-inflammatory disease, self-limited acute febrile attacks, inflammation of serosa and synovial, and associated with arthritis, arthralgia, myalgia, and erysipeloid-like rash, pericarditis, scrotal pain, aseptic meningitis, thrombosis, and vasculitis, pleuritis)	Loss-of-function mutations in NLRP3 and NLRP6 MEFV genes	[106]
CAPS	**NLRP3 and NLRP6** (cutaneous and systemic, musculoskeletal, and central nervous system inflammation)	Gain-of-function mutation	[106, 107]
Graft-versus-host disease	**NLRP6** (complication of allo-HCT associated with intestinal dysbiosis)	NLRP6 activation aggravates allogeneic GVHD (unknown mechanism)	[108]
Colitis	**NLRP6** (inflammation of the inner lining of the colon)	Aberrant activation and dysfunction of NLRP6	[109, 110]
FBHM	**NLRP7** (recurrent abnormal pregnancy with no embryonic development and cystic degeneration of placenta villi)	Recessive mutation in NLRP7	[111]
Growth retardation	**NLRP7** (DNA methylation, trophoblast hypertrophy)	Low level of NLPR7 in trophoblasts	[112]

*COPD* severe chronic obstructive pulmonary disease*. MSPC* multiple self-healing palmoplantar carcinomas*. KAs* keratoacanthomas (which include Muir-Torre syndrome, Witten-Zak syndrome, Grzybows-ki syndrome, and multiple self-healing squamous epitheliomas)*. SLE* Systemic lupus erythematosus*. JRRP* juvenile-onset recurrent respiratory papillomatosis*. FKLC* familial keratosis lichenoides chronica*. FCAS* familial cold auto-inflammatory syndrome*. FBHM* familial biparental hydatidiform mole. *FMF* familial Mediterranean fever. *MEFV* Mediterranean fever*. MEFV gene* a human gene controlling production of pyrin, a component of NLRPs. *MWS* Muckle-Wells syndrome*. Aβ* amyloid-β. αSyn, α-synuclein*. CPPD* calcium pyrophosphate di-hydrate*. CAPS* Cryopyrin-associated periodic syndrome*. EAE* experimental autoimmune encephalomyelitis*. MCA* methyl-cholanthrene. *MS* multiple sclerosis*. ROS* reactive oxygen species*. GVHD* graft-versus-host disease

When the rare gain-of-function mutations on the PYD or LRR domain of NLRP1 were described in 2016, the NLRP1 stepped into the spotlight in skin-related inflammatory pathologies [43]. Notably, MSPC patients carry inherited mutations within the N-terminal PYD domain (A54T, A66V and M77T), and patients with FKLC display an in-frame deletion (F787-R843) in the LRR domain. As expected, these mutations were confirmed to perturb this auto-inhibitory activity because MSPC mutations disrupt PYD folding and FKLC deletion may weaken NLRP1 auto-inhibitory function. Thus, the PYD and LRR domains are thought to play an auto-inhibitory role in NLRP1.

Furthermore, L155H and M1184V are two polymorphisms of NLRP1 that will increase the risk for vitiligo disease. Mechanistically, M1184V causes a significantly increased processing of pro-IL-1β by caspase-1 in the reconstituted HEK293T system, suggesting a potential disease-associated molecular mechanism [45]. The T755N mutation of NLRP1, located within the linker between the NACHT and LRR domain, resulted in a syndromic named juvenile-onset recurrent respiratory papillomatosis (JRRP) [104]. Auto-inflammation with arthritis and dyskeratosis (AIADK) patients who displayed skin lesions, polyarthritis and periodic fever with increased caspase-1 and IL-18, carry R726W and P1214R mutations [113]. The P1214R mutation is close to the cleavage site of the NLRP1 FIIND domain and abolishes NLRP1-DPP9 interaction to result in the auto-activation of NLRP1 and subsequent inflammasome signaling [87]. Furthermore, gain-of-function mutations in lung NLRP1 have been associated with occurrence of a rare upper airway inflammatory disease caused by the human papilloma virus [104]. However, the molecular mechanisms that most mutations of NLRP1 lead to these auto-inflammatory diseases are required to investigate the detailed role of NLRP1 further.

## CARD8 inflammasome: an nlrp1 analogous inflammasome

### CARD8 inflammasomes and its role in health and innate immunity

Recent breakthroughs and in-depth studies have demonstrated the existence of an NLRP1 inflammasome-like hetero-multimeric complex protein forming an inflammasome, also known as NLRP1 analogous inflammasome or CARD8 inflammasome. The discovery of the CARD8 inflammasome occurred during characterization of the pyroptosis-inducing activity of the non-selective dipeptidyl-peptidase (DPP)-inhibitor Val-boroPro (VbP, Talabostat) and its associated compounds. Indeed, while it is noteworthy that VbP triggers caspase-1-associated pyroptosis [114, 115] via activation of human NLRP1 [86] or mouse NLRP1B [87], it has been also found that upon VbP treatment in human keratinocytes, CARD8 could trigger pyroptosis. Specifically, after inhibition of DPPs using VbP in human myeloid leukemia cells, NLRP1 was found intact and inactive while a CD4^+^ and CD8^+^ T-cell death process, characterized as pyroptosis, occurred. Finally, it was demonstrated that this pyroptosis depends on the CARD8-caspase-1-GSDMD-associated pathway and only occurred in resting but not in active T-cells [116]. Of interest, unlike NLRP1 inflammasome, which is present in both human and murine systems (to a lesser extent), CARD8 inflammasome has only been identified in humans and not in murine systems [117, 118]. CARD8 inflammasome uses the FIIND domain and its CARD domain as sensors to directly interact with and activate caspase-1 [61, 116], and has been mainly evidenced from HIV-1 infection-associated inflammatory response [119].

### Recent breakthroughs in CARD8 inflammasome

CARD8 is the only other human inflammasome mediator highly similar to NLRP1, with highly similar domain organization that includes the FIIND domain with the self-cleavage site and carboxyterminal CARD domain (Figs. 1 and 3). The structured N-terminal PYD, NOD and LRR domains of NLRP1 are replaced by a disordered N-terminal region in CARD8. The auto-proteolytic activity of FIIND domain of CARD8 results in a non-covalent association between N- and C-termini of CARD8, similar to what has been found in NLRP1 inflammasome. The N-terminal fragment of CARD8 can be degraded by a functional degradation model, and the bioactive C-terminal UPA-CARD of CARD8 is used to form an inflammasome, directly interacting with the CARD of proCaspase-1 for inflammasome activation. For CARD8 and NLRP1, the FIIND domain associates with DPP8/9 to sequester the bioactive component in a ternary complex for restricting the spontaneous inflammasome activation [88, 89, 120] (Fig. 3c). Furthermore, the CARD8 T60 variant is found that it directly interacts with NLRP1 to act as a negative regulator to control the NLRP1 inflammasome activation level [121].

CARD8 and NLRP1 are the tripwire sensors that are activated by pathogen-encoded activities. The human immunodeficiency virus 1 (HIV-1) protease could trigger CARD8 inflammasome assembly to activate caspase-1 and secret IL-1β that resembles NLRP1 [119]. In this regard, HIV protease cleaves N-terminal fragment between phenylalanine (F) 59 and F60 to expose a destabilizing N-degron that can be ubiquitylated for degradation machinery. Subsequent proteasome-mediated degradation of the N-terminal protein fragment removes the self-inhibition function of CARD8 and liberates the C-terminal UPA-CARD to assemble the CARD8 inflammasome. Several studies have reported that the usage of non-nucleoside reverse transcriptase inhibitors (NNRTIs) could lead to protease activity of HIV to kill infected cells, which is due to the activation of CARD8 inflammasome [122].

Furthermore, under DPP9 inhibitors treatment conditions, CARD8 inflammasome will reduce the activation threshold to effectively clear the HIV-infected cells [123]. Additionally, CARD8 could act as an important immune sensor of infection by positive-sense RNA viruses, including *Coronaviridae*, *Picornaviridae* and *Retroviridae* [124]. For the detailed mechanism, the 3CL protease encoded by these RNA viruses could cleave the unstructured N-terminal region of CARD8, leading to the release of C-terminal CARD-containing fragment that is sufficient for inflammasome assembly. DPP8/9 inhibitors, VbP, accelerate the degradation of CARD8 to destabilize the repressive ternary complex for CARD8 inflammasome activation [120]. Structural and biochemical studies of the ternary complex revealed that CARD8 and NLRP1 directly interact with DPP8/9, but only the neo-N terminus of NLRP1 binds to the DPP8/9 active site. VbP could disrupt this interaction between NLRP1 and DPP8/9, but not CARD8 and DPP8/9, to activate human NLRP1 and CARD8 inflammasome [88, 89, 120, 125]. CARD8 inflammasome was found to be required for VbP-induced pyroptosis in human macrophages and resting lymphocytes expressing more CARD8 than NLRP1, and NLRP1 inflammasome is indispensable for VbP-induced cell death in skin and airway epithelial cells with high expression of NLRP1 [116–118].

The danger-related signals detected by CARD8 inflammasome have not yet been fully established. A recent study reported that the protein fold disruption could induce proteasome-mediated degradation and cause cytosolic peptide accumulation, destabilizing the CARD8-DPP8/9 ternary complexes to activate the CARD8 inflammasome [75]. On the other hand, the M24B aminopeptidases have been identified to regulate the CARD8 inflammasome activation recently [126]. The M24B aminopeptidases prolidase (PEPD) and X-prolyl aminopeptidase 1 (XPNPEP1) could catabolize peptides named Xaa-Pros that contain a P2 proline (Xaa is any amino acid). When PEPD/XPNPEP1 is inhibited, the accumulation of Xaa-Pros will weakly inhibit DPP8/9 activity, selectively activating the CARD8 inflammasome but not the related NLRP1 inflammasome. The other danger signal that could activate CARD8 inflammasome is reductive stress. A recent study characterized that a radical-trapping antioxidant, JSH-23, induces reductive stress and accelerates the N-terminal degradation of CARD8 [77]. The radical-trapping antioxidant works synergistically with VbP to induce more pyroptotic cell death and inflammatory cytokine secretion. In recent years, many advances in the CARD8 field propelled our understanding of its function, but future studies are needed to determine the more detailed molecular mode of action of CARD8.

## NLRP3 inflammasome

### NLRP3 inflammasome and its role in health and innate immunity

Primary localized in the microglia, NLRP3 is another member from the NLRP family, discovered to be associated with and form inflammasome after NLRP1 inflammasome, but is the first to be extensively described and well-characterized amongst the canonical NLRP inflammasomes. Described for the first time in human brain, NLRP3 inflammasome consists of NLRP3, ASC, and pro-caspase-1; its detailed structure is described herein. Unlike NLRP1 inflammasome, NLRP3 inflammasome senses a wider variety of activator/stimuli (including TLR agonists [LPS, nigericin, monosodium urate crystals, and ATP], pathogens [fungi, bacteria, and viruses], pro-inflammatory cytokines [tumor necrosis factor, TNF], intracellular components [reactive oxygen species, ion flux, lysosomal disruption-, mitochondrial dysfunction-, metabolic changes and trans-Golgi catabolism-associated components]) [3, 23, 127, 128]. In microglia, NLRP3 inflammasome is activated by proteins such as misfolded or aggregated amyloid-β, α-synuclein and prion protein or superoxide dismutase [129], and members of the complement pathway, and induces production of IL-1β and IL-18 [130]. The NLRP3 inflammasome has been found to be involved in almost all aspects of health and diseases (Fig. 2 and Table 1). For instance, in the majority of health threats, including auto-inflammatory, metabolic, neurodegenerative, and some infectious diseases [128], expression of NLRP3 has been found to be increased alongside with high levels of IL-1β, and IL-18 production, which has attracted an impressive interest for research. Thus, this has been the main reason that justifies its deep and well characterization.

### Structural and functional organization of NLRP3 inflammasome

The NLRP3 inflammasome is a multiprotein complex mediating the secretion of proinflammatory cytokines IL-1β and IL-18 and inducing inflammatory cell death (pyroptosis). Also known as NALP3, cryopyrin, PYPAF1, CIAS1, and CLR1.1, the NLRP3 inflammasome has been so far the first extensively and best-characterized canonical inflammasome of the NLRP inflammasomes. NLRP3 inflammasome has been named after its main protein (the NLRP3), which acts like the sensor of the inflammasome, and complexed with two other proteins, including the apoptosis-associated speck-like protein containing a caspase-recruitment domain (ASC) serving as the adaptor, and the enzyme pro-caspase-1 serving as the effector. Specifically, each component of the NLRP3 inflammasome contains active domains playing crucial roles in the activation and functions of NLRP3 inflammasome. The structure of NLRP3 protein contains 3 active domains, including a central nucleotide-binding and oligomerization (NACHT, aka NOD) domain, flanked by an N-terminal pyrin domain (PYD) domain and a C-terminal leucine-rich repeat (LRR) domain. The central NACHT domain mediates nucleic acid ligation and promotes protein oligomerization; the N-terminal PYD domain is involved in the association of NLRP3 and caspase-1 through interaction with ACS protein; the C-terminal LRR domain is involved in recognition and binding of the inflammasome to putative ligands, including PAMPs and DAMPs, respectively, thus facilitates activation of the NLRP3 inflammasome. The adaptor ASC protein of the NLRP3 inflammasome consists of two domains, including an N-terminal PYD and a C-terminal caspase recruitment (CARD) domain, from which the name PYCARD was attributed. It promotes the binding of NLRP3 (through homotypic PYD-PYD interaction) and pro-caspase-1 (through homotypic CARD-CARD interaction). The enzyme pro-caspase-1 is also a two-domain protein, which consists of a CARD and a caspase domain, containing two sub-units p20 and p10 that act as a catalytic domain. The p20 is the central large catalytic subunit while p10 is the C-terminal small catalytic subunit [106, 128, 131, 132] (Fig. 1).

**Fig. 5:**
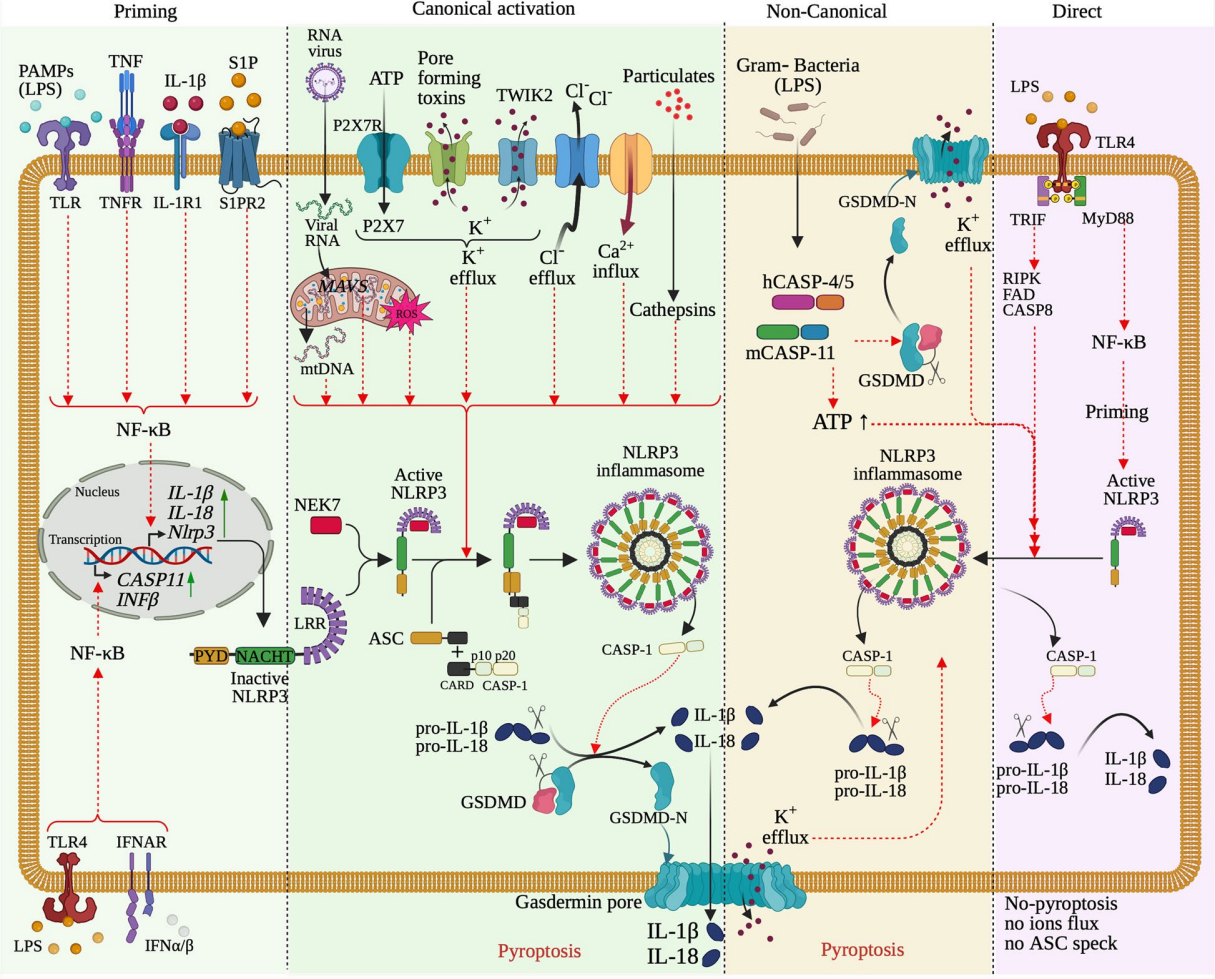
The mechanisms of human NLRP3 inflammasome activation and regulation. The activation of NLRP3 inflammasome occurs either through a canonical two-step pathway or a non-canonical pathway, and a direct or alternative pathway. The canonical activation pathway involved 2 steps: a priming (signal 1, left panel) and an activation (signal 2, second panel from left) steps. Priming step is induced by NLRP3 signals, including LPS and TNF, IL-1b, IFNs, lipopolysaccharide (LPS), and sphingosine-1 phosphate (S1P), activate NF-κB that; in turn upregulates the transcription of *Nlrp3* gene and other genes (ASC and pro-caspase1) involved in NLRP inflammasome, by interacting with and triggering their receptors. Once transcribed, NLRP3 is pre-activated by interacting with NEK7, forming a complex that will be activated into hetero-complex inflammasome. The canonical activation of NLRP3 inflammasome is induced by signal 2 including PAMPs (nigericin, viral RNA, and MDP) and DAMPs (extracellular ATP, mtDNA, and mtROS) and particulates. The molecular mechanisms behind the polymerization and the activation of NLRP3 inflammasome include activation of several signaling events, including induction of K^+^ efflux, Ca^2+^ flux, Cl^–^efflux, lysosomal disruption, mtROS production, and release of oxidized mtDNA in the cytosolic compartment. Thus, formation of NLRP3 inflammasome includes oligomerization of NLRP3-NEK7, recruitment of ASC, and Casp1. auto-proteolysis of proteolytic cleavage of Casp1 releases p10/p20 active enzyme, which digest Pro-IL-1*β* and Pro-IL-18 into IL-1*β* and IL-18 cytokines to promote proinflammatory responses. The subunit p10/p20 of Casp1 also digests GSDMD releasing GSDMD-N that form cell membrane pore to result in pyroptosis of the cell. Non-canonical activation of NLRP3 inflammasome (third panel from left) occurs without priming, as Casp4 is already present in the cytoplasm, and is induced by gram-negative bacteria that release LPS into the cell cytosol. Released LPS activates Casp11 in human (and Casp4/5 in mouse), which cleaves GSDMD complex releasing GSDMD-N that forms gasdermin pores and induces pyroptosis. The gasdermin pore formed constitutes a channel for K^+^ efflux, which activates the NLRP3 inflammasome, and consequently activate Casp1 and IL-1*β* and IL-18. The alternative pathway (right panel) activation is induced by TLR4 agonists that activates the TLR4-TRIF-RIPK1-FADD-Casp8 signaling pathway. Consequently, Casp8 activates the NLRP3 inflammasome. Note that, there is no need of K^+^ efflux, ASC speck formation, to activate inflammasome, and there is no pyroptosis

Upon stimulation, a cascade of protein–protein interactions occurs and ends up in the formation of the active NLRP3 inflammasome complex (Fig. 5). Specifically, the upstream signals activating the NLRP3 inflammasome induce oligomerization, a typically thought conformational changes of NLRP3 protein. The oligomerized NLRP3 in turn uses its N-terminal PYD domain to recruit the adaptor ASC protein through a homotypic PYD-PYD interaction with the N-terminal PYD domain of ASC and nucleates helical ASC filament formation. Subsequently, the adaptor ASC protein, in the form of a complex of fused multiple ASC filaments (aka ASC speck) [56, 128, 129], uses its C-terminal CARD domain to recruit the enzyme pro-caspase-1 through a homotypic CARD-CARD interaction with the N-terminal CARD domain of the effector. Interestingly, extensive studies on the structural organization and components of NLRP3 inflammasome have revealed that NIMA-related kinase 7 (NEK7), a serine-threonine kinase involved in mitosis, also interacts with NLRP3 and contributes to NLRP3 inflammasome activity, thus is an entire component of the NLRP3 inflammasome [133–135]. Specifically, from the upstream activation steps, NEK7 binds to and oligomerizes together with NLRP3. This oligomerized complex is essential in recruiting the adaptor ASC protein, but especially favoring ASC speck formation, inducing nucleates helical ASC filament formation, and caspase-1 activation [134, 135]. The NLRP3–ASC–Pro-Capase-1 multiprotein oligomeric complex is the active form of NLRP3 inflammasome, which mediates the proximity-related self-cleavage of pro-caspase-1 to generate the active caspase-1. Then, the catalytic active subunit p20/10 is released from the self-cleavage in the form of heterotetramer, which accomplishes the enzymatic activity of caspase-1, including activation of specific pro-inflammatory cytokines, including pro-IL-18 and pro-IL-1β into IL-18 and IL-1β, their biologically active mature form [136, 137] (Fig. 5 [132]). Upon IL-18 and IL-1β cytokine released, the active subunit p20/10 is degraded, as it is instable in cell cytosolic environment [106, 128, 131, 132, 136].

### Molecular mechanism of NLRP3 inflammasome activation

The NLRP3 inflammasome is highly expressed in human cells that contribute to the immune defense pathogenesis including macrophages, monocytes, neutrophils, dendritic cells, and lymphocytes, but also in non-immune cells, including endothelial cells, cardio-myocytes, fibroblasts, osteoblasts, and epithelial cells [106, 128, 131, 132, 136, 138, 139].

Even though NLRP3 inflammasome is the best-characterized canonical inflammasome, the intracellular upstream mechanisms and stimuli activating this inflammasome are not well-defined [140]. Nevertheless, many studies proposed several structurally and chemically different stimuli involved in the upstream activation steps of NLRP3 inflammasome. These stimuli include PAMPs, DAMPs, ionic (potassium [K^+^], chloride [Cl^–^], and calcium [Ca^2+^]) flux, reactive oxygen species (ROS) produced after mitochondrial dysfunction (mtROS), lysosomal damages, metabolic changes, and trans-Golgi disassembly-associated particles [7, 128, 131]. Moreover, there are no concluding studies clearly demonstrating a direct interaction between at least one of the aforementioned stimuli and a component of the NLRP3 inflammasome. Saying that, the clear mechanism initiating NLRP3 inflammasome activation still remains hypothetical, thus to be deeply investigated and confirmed. However, it is thought that NLRP3 senses common cascade cellular events induced by these proposed stimuli.

Early studies have proposed a two-signal model for NLRP3 inflammasome activation. Indeed, the first priming signal of NLRP3 inflammasome response is to induce the expression of NLRP3 inflammasome protein components because of low expression levels in a variety of cell types [141–143]. The interaction between extracellular PRRs and PAMPs/DAMPs induces the transcriptional activity of intracellular inflammatory signaling molecules, such as nuclear factor (NF)-kB and activator protein-1 (AP-1) to upregulate the production level of NLRP3 inflammasome molecules [144]. The next triggering signal is initiated to assemble NLRP3 inflammasome complexes and result in the signal transduction cascades to ultimately active caspase-1. Enzymatically active caspase-1 cleaves the pro-inflammatory cytokine (IL-1β and IL-18) to yield bioactive cytokine and naturally, autoinhibited gasdermin-D (GSDMD) to release the cellular content into the extracellular space by membrane pores formation [145–149]. Besides, recent studies proposed two other models of activation mechanism; the non-canonical and the single-step NLRP3 inflammasome activation models (review in [128]).

#### The two-step NLRP3 inflammasome activation model

The two-step (or two-signal) NLRP3 inflammasome activation model, also known as the canonical activation model, consists of a first step (hereafter subtitled “A-signal 1”) highly regulated and required to trigger oligomerization of NLRP3. Specifically, NLRP3 is sensed by a first signal through recognition of PAMPs and DAMPs released by microbial components or endogenous cytokines. This first event is known as the “priming” of NLRP3 inflammasome activation (Fig. 5, left panel). The second step (hereafter subtitled “B-signal 2”), similar to an effector step, is required to terminate the formation of the active NLRP3 inflammasome complex, hence the name “activation step” given to this signal. This second signal is a following cascade reaction of the priming step activated by NLRP3 stimuli and can be triggered through exchange with the extra- or intracellular space components that include extracellular ATP, pore-forming toxins, K^+^ and Cl^–^ efflux, Ca^2+^ influx, RNA viruses, and particulate matter, which activate the NLRP3 inflammasome [128, 131, 140, 150, 151] (Fig. 5, second panel from left).

### A- Signal 1: Priming the NLRP3 inflammasome

Inflammation is a protective immune response, specifically an inflammatory process triggered upon infection, thus, it needs to be regulated (induced in presence of antigens and repressed after infection clearance), to avoid and prevent dysfunction-associated diseases. The priming step of the NLRP3 Inflammasome lies on the up-regulation and induction of the transcriptional level of the pro-IL-1β (precursor of IL-1β) and NLRP3 proteins, but not the ASC and pro-caspase-1 proteins. In fact, it is noteworthy that at the homeostasis, NLRP3 and pro-IL-1β are generally present but low or not detected, respectively, in macrophages and most of the effector cells of innate immune system [141–143, 152], making them unable to initiate a protective inflammatory response. The transcriptional up-regulation starts upon exposition of macrophages to priming stimuli (also known as priming signals). The priming stimuli include PAMPs/DAMPs or cytokines (tumor necrosis factors [TNFs] and pro-IL-1β), which bind to and activate their respective cell surface receptors, including TLRs (such as PRRs and NODs) or cytokine receptors (such as TNF-α receptors [TNFRs] and IL-1 receptors [IL-1R]). These extracellular host–pathogen interactions subsequently activate the nuclear factor-kappaB (NF-κB) signaling pathway, which in turn, induces transcriptional up-regulation and the expression of NLRP3, pro-IL-1β, and pro-IL-18. It is important to note that when the TLRs are excited by PAMPs/DAMPs, the NF-κB signaling pathway up-regulates the induction of NLRP3 and pro-IL-1β through both MyD88 (myeloid differentiation primary response 88) and TRIF (TIR-domain-containing adapter-inducing interferon-β) that serve as signaling molecules of NF-κB signaling pathway [141].

Furthermore, both apoptotic signaling molecules caspase-8 and FADD (FAS-associated death domain) have been found to be required for NLRP3 transcription induction during the priming step (reviewed in [106, 131]), as well as the cytoplasmic NOD1/2 [141]. Indeed, Caspase-8 interacts with the kinase inhibitor complex of NF-κB, then promotes its induction of NF-κB transcription and translocation [153], while FADD, with its dual role in the NF-κB signaling pathway, can either induce activation of NF-κB signaling pathway during priming or, by promoting apoptosis, repress NF-κB activation [154]. Besides, TLR4 ligand lipopolysaccharides (LPSs) are also involved in priming the NLRP3 Inflammasome. The TLR4 ligand LPSs induce a metabolism shift in macrophages from oxidative phosphorylation to glycolysis, indirectly stabilizing the hypoxia-inducible factor 1α (HIF1α) and up-regulating transcription of IL-1β gene [155].

### B- Signal 2: Activating the NLRP3 Inflammasome

In general, the priming step prepares the main NLRP3 inflammasome components for the formation of the active multiprotein oligomeric complex known as NLRP3 inflammasome, as described previously. The “signal 2” which includes or englobes the entire different mechanistic steps, is meant to promote or induce this structurally organized assembly, which, once activated, promotes the maturation and release of IL-1β and IL-18, thus triggering a downstream inflammatory response (pyroptosis) [156]. In the meantime, the activated caspase-1 cleaves the gasdermin D (GSDMD), a pro-pyroptosis protein that forms transmembrane pores and favors the release of mature IL-1β and IL-18 yielding a strong inflammation and cell death (pyroptosis) [157].

Generally and as aforementioned, in non-stimulated macrophages, the NLRP3 and pro-IL-1β proteins already exist in macrophages but in latent form at a low concentration [141–143, 152]. The priming signal increases the transcription and expression level of NLRP3 into its inactive but activation-competent state (same as for IL-1β), which is then activated by a wide range of distinct stimuli (signals). It has been widely considered that NLRP3 inflammasome activation pathway lies on three mechanisms, including intra- and extracellular ionic flux (K^+^ and Cl^–^ efflux, Ca^2+^ influx), reactive oxygen species (ROS) mainly but not only produced after mitochondrial dysfunction (mtROS), and lysosomal damages [128, 131, 140, 150, 151] (Fig. 5).

Although there are many available studies that described and reviewed the upstream activation mechanisms of NLRP3 inflammasome, some data remain unclear, conflicting, or confusing. We briefly recalled the known mechanisms but emphasized conflicting or hidden mechanisms disclosing the latest findings.

### Ionic flux: K^+^ and Cl^–^ efflux, Ca^2+^ influx

The “priming” stimuli not only up-regulates cytosolic transcriptional levels of pro-IL-1β and NLRP3, but its NLRP3 up-regulation triggering effect also induces upstream events responsible for NLRP3 inflammasome activation, including ionic flux events, in response to the cell threats. These ionic events include K^+^ and Cl^−^ efflux, Ca^2+^ distribution, and N^a+^ influx.

Cytosolic potassium ion (K^+^) efflux or depletion (decrease of intracellular K^+^) has long been considered a common stimulating event for NLRP3 inflammasome activation. Specifically, the NLRP3 stimuli stimulate extracellular ATP, which induces intracellular K^+^ efflux via the TWIK2 (two-pore domain weak inwardly rectifying K^+^ channel 2) [158], in coordination with P2X7 (the purinergic ion channel receptor type 2, family X, subunit 7), to maturate IL-1β and activate NLRP3 assembly [128, 159, 160]. It is important to note that, although P2X7 is involved in maturation of IL-1β and coordinates with TWIK2 [161], it is not an ionic channel for K^+^ efflux [162], but TWIK2 is (Fig. 5). Furthermore, while how K^+^ is activated was unknown, a recent study by Huang et al*.* [161] found a mechanism by which K^+^ efflux is activated and demonstrated that Rab11a (a Ca^2+^–sensitive GTP-binding protein) plays a central role in K^+^ efflux-based NLRP3 inflammasome activation. Specifically, the increase extracellular ATP also induces endosomal TWIK2 plasmalemma trafficking, which is regulated by Rab11a, such that Rab11a deletion prevents endosomal fusion with the plasmalemma and K^+^ efflux, and therefore prevents activation of NLRP3 inflammasome in macrophages. Besides, With-No-Lysine (WNL or WNK) kinase signaling pathway has been found to be a master regulator and controller of intracellular K^+^ balance. Specifically, upon cell threat caused by stress or osmotic changes, WNL kinase activates SPAK (Ste20-related Proline-Alanine rich Kinase) and OXSR1 (Oxidative Stress Response Kinase 1) kinases which block KCC channels (K-2Cl cotransporter, which pump K^+^ out of cells), and promote NKCC channels (Na^+^-K^+^-2Cl^–^ cotransporter, which pump K^+^ inside cells) [163].

However, how NLRP3 senses the K^+^ intracellular decrease remains unclear. To attempt to understand the NRLP3 sensing mechanism, some recent studies suggested that K^+^ efflux might be followed by a downstream mechanism that directly interacts with and induces NLRP3 oligomerization [131]. Moreover, supporting studies corroborate this hypothesis, and found that the newly identified NLRP3-binding protein NEK7 would sense and act downstream K^+^ efflux to regulate both its own and NLRP3 oligomerization and activate NLRP3 inflammasome through a bridging *NLRP3* protomer-mediated direct NEK7-NLRP3 interaction [164, 165]. In absence of NEK7, activation of caspase-1 and release of IL-1β were abolished [134, 165], suggesting that NEK7 might be a downstream K^+^ efflux sensor that activates NLRP3 [164].

Besides K^+^ efflux, sodium/chlorine redistribution, specifically Na^+^ influx and Cl^−^ efflux are ionic events involved in NLRP3 inflammasome complex activation. Contrary to K^+^ efflux which is enough and can induce activation of NLRP3 alone, it has been demonstrated that Na^+^ influx is unable to activate NLRP3 inflammasome [151], but acts much more like a regulator in NLRP3 inflammasome activation, possibly by regulating the K^+^ efflux induced by NLRP3 stimuli [131]. Similarly, while Cl^–^ efflux has been found to induce ASC speck formation (nucleates helical ASC filament formation, and caspase-1 activation [134, 135]), it cannot induce NLRP3 inflammasome activation without K^+^ efflux [166]. As previously mentioned, WNL kinase signaling pathway regulates cellular Cl^–^ balance by acting on chloride channels. Through phosphorylation, WNL kinase activates SPAK and OXSR1 kinases, which in turn activate chloride channels such as NKCC channels (Na^+^-K^+^-2Cl^–^ cotransporter) [163]. Chloride channels, including the volume-regulated anion channel (VRAC), chloride intracellular channels (CLICs) or NKCC, trigger Cl^–^ efflux, which modulate NLRP3 inflammasome activation [167–169]. Specifically, in response to mitochondrial dysfunction, CLIC translocation to plasma membrane triggers Cl^–^ efflux, which promotes NLRP3-NEK7 interaction [163, 167, 170] and consequently induces NLRP3 inflammasome activation. Taken together, Na^+^ influx and Cl^−^ efflux cannot act on their own but must coordinate with K^+^ efflux to activate NLRP3 inflammasome.

While involvement of Na^+^, K^+^ and Cl^–^ have been well described in NLRP3 oligomerization, the role of Ca^2+^ in NLRP3 inflammasome activation is controversial. Ca^2+^ has been proven to contribute in many bimolecular processes including in NLRP3 inflammasome activation [171]. On the first hand, numerous studies support that Ca^2+^ flux is an upstream event that triggers NLRP3 oligomerization and IL-1β release, in response to cell threats and NLRP3 stimuli. Indeed, earlier studies showed that inhibition of Ca^2+^ production prevented IL-1β secretion [172, 173], suggesting that Ca^2+^ plays a central role in NLRP3 inflammasome activation. In addition, studies supporting Ca^2+^ crucial role have been well documented (reviewed in [128, 131]). On the other hand, strong evidence demonstrated that Ca^2+^ is not involved in NLRP3 inflammasome activation, but is a consequence of the NLRP3 activation. In other words, Ca^2+^ mobilization occurs downstream of both NLRP3 and caspase-1 activation [174]. Thus, there is a need to determine the role of upstream mobilization of Ca^2+^ in NLRP3 inflammasome activation and how post-NLRP3 activation Ca^2+^ flux affects the downstream events of NLRP3 inflammasome activation.

### Mitochondrial dysfunction-associated ROS (mtROS)

ROS – and other debris (including DNA and cardiolipin) – produced through mitochondrial dysfunction or dynamic are other upstream activating events proposed amongst the signal 2 to be involved in activating NRLP3 inflammasome [175, 176]. As a reminder, for these proposed events, the NLRP3 inflammasome activation is triggered by the increased level of mtROS and mtDNA, because in normal cell homeostasis, mitochondria continuously produce ROS as a product of the electron transport chain but in small and cell tolerable titer. Whereas mitochondrial fission induces high production of mtROS and mtDNA, responsible for NLRP3 activation [177]. Several studies demonstrated that, in response to mitochondrial damages and/or NLRP3 stimulating activators, released mtDNAs are oxidized by the overexpressed cytosolic mtROS, which in turn activate NLRP3 inflammasome during apoptosis thanks to a direct oxidized mtDNA-NLRP3 interaction [175, 178–180]. Specifically, it is thought that overexpressed mtROS-based NLRP3 inflammasome activation is due to a two-signal model, including NF-κB signaling pathway and NLRP3 ligand mitochondria/thioredoxin-interacting protein (TXNIP) [181]. In the former pathway, activation of NF-κB signaling pathway would induce transcriptional up-regulation and the expression of NLRP3, pro-IL-1β, and pro-IL-18 like during the priming. The latter pathway is activated upon ROS accumulation, and promotes the binding of circulating TXNIP and oxidized mtDNAs to NLRP3 protein, which yield activation of NRLP3 inflammasome [181].

Besides the generation of mtROS and mtDNA, mitochondria and NLRP3 inflammasome components are shown to be co-localized, such that upon mitochondria damage or dysfunction, mitochondria release debris molecules, including cardiolipin, mitochondrial antiviral-signaling protein (MAVS), and mitofusin 2, which bind to NLRP3 inflammasome components in response to NLRP3 stimuli, facilitating the multi-protein complex assembly. Thus, mitochondria have been also considered as a bridging site for NLRP3 inflammasome assembly. For instance, upon virus RNA, extracellular ATP, and nigericin, but no other NLRP3 stimuli, the direct interaction of MAVS with NLRP3 was observed and demonstrated to be required, suggesting that MAVS contributes to NRLP3 inflammasome assembly [182]. Specifically, Guan et al [183]. reported that MAVS promotes NLRP3 inflammasome activation by targeting ASC for K63-linked ubiquitination via the E3 ligase TRAF3. A recent report demonstrated that extracellular ATP-, nigericin-, or LPS-associated MAVS expression activates polyribonucleotide nucleotidyltransferase 1 (Pnpt1), which mediates NLRP3 inflammasome-dependent IL-1β release [184].

The roles of mitochondrial mitofusin 2 [185] and cardiolipin [186] in interacting with and triggering NLRP3 inflammasome component assembly were demonstrated. More specifically, upon TLR4 agonists, cardiolipin, a mitochondrial intermembrane space lipid, is found to be released in the cytosol and could directly bind to the LRR domain and caspase-1 domain of NLRP3 inflammasome, as a knockdown of cardiolipin expression inhibits NLRP3 activation [186, 187]. However, the clear role of mitochondria and their released debris should be deeply investigated and confirmed, since there are studies showing that different cell components, including ASC pyroptosome but not mitochondria are involved in assembly of NLRP3 inflammasome (review in [128, 131]).

### Lysosomal damages

Particulate matters released in the cytosolic environment after lysosomal disruption, have been reported as one of the up-stream events that activate NLRP3 inflammasome, and consequently, trigger a protective inflammation [128, 131]. Indeed, after pathogen infection, non-self molecule, or particulate matter recognition, innate cells engage in phagocytosis as an elimination process. Specifically, particulate matters, including self-derived particulates such as uric acid, amyloid-β, calcium crystals, and cholesterol crystals, or foreign-derived particulates such as aluminium salts, silica crystals, and asbestos are engulfed into a phagosome and later form a phagolysosome [188]. Acidification of lysosome causes phagolysosomal swelling, which results in lysosomal disruption and release of lysosomal content within the cytosol. Note that lysosomal disruption is induced by the lysosomotropic dipeptide Leu-Leu-OMe [188], and lysosomal released content is found to be a critical step for NLRP3 inflammasome activation by particulate matter. Even though the mechanism describing how lysosomal disruption and released lysosomal contents induce NLRP3 inflammasome activation remains unclear, it has been demonstrated that cathepsins present within the lysosomes are involved in NLRP3 activation in response to particulate matters. In fact, genetic deletion and experimental inhibition or knockdown of cathepsins B, X, L, or S prevent activation of NRLP3 inflammasome [189]. Furthermore, it was reported that release of lysosomal cathepsin is required for IL-1β induction, which suggests that cathepsin X, L, S, but specifically cathepsin B in involved in NLRP3 inflammasome activation [189–192]. Besides, many studies reported that lysosomal disruption and lysosomal released particulate matters induced by the lysosomotropic dipeptide Leu-Leu-OMe, have a stimulant effect on ionic flux, including K^+^ efflux, Cl^–^ efflux, and Ca^2+^ influx, which suggests that ionic efflux, especially K^+^ efflux might be the central hallmark in NLRP3 inflammasome activation pathways [151, 193, 194].

#### The non-canonical NLRP3 inflammasome activation model

In addition to the canonical activation model that lies on priming and caspase-1 activation as previously described, there is also a non-canonical activation model of NLRP3 inflammasome, which lies on the non-canonical caspase-4 and caspase-5 direct activation by intracellular stimuli. In mice, the non-canonical NLRP3 inflammasome activation model is mediated by activation of caspase-11 [195]. Priming is not necessary in this activation pathway and the physiological defense system activates this pathway mainly when pathogens have bypassed cell surface TLR4 [196]. Of interest, in infections by and during phagocytosis of only gram-negative but not gram-positive bacteria, lipopolysaccharides (LPSs) released in the cytosol of phagocytes are sensed by, directly bind to, and stimulate human caspase-4 and caspase-5 or mice caspase-11 (independently of TLR4), resulting in the oligomerization and auto-cleavage-based activation of the caspases, and induces the non-canonical activation of NLRP3 inflammasome. In turn, active human caspase-4 and caspase-5 or murine caspase-11 induces cleavage of GSDMD (an N-terminal-associated membrane forming pore pro-pyroptosis protein) and favors the efflux of potassium ions (K^+^) (through ATP release), activation and oligomerization of NLRP3, formation of ASC specks, and release of mature IL-1β and IL-18, consequently yielding a strong inflammation and cell death (pyroptosis) [27, 157].

#### The one-step NLRP3 inflammasome activation model

The one-step NLRP3 inflammasome activation model, aka the direct NLRP3 inflammasome activation model, has been described as an alternative activation of NLRP3 inflammasome. Many studies revealed that in certain circumstances, phagocytes, stimulated by LPSs do not require a second activation, neither do they need activation of caspase-1 before they can release mature IL-1β and IL-18, which activate NLRP3 inflammasome [197–199]. This NLRP3 inflammasome activation mechanism is quicker than the formers and occurs through the TLR4-TRIF-RIPK1-FADD-CASP8 pathway. Specifically, unlike canonical and non-canonical NLRP3 inflammasome activation mechanisms described here before in which caspase-1 and caspase-4/5 are respectively required to promote the release of mature IL-1β and IL-18, K^+^ efflux, and formation of ASC specks in human, the one-step activation pathway lies on caspase-8, which alternatively cleaves IL-1β and IL-18, either directly or through the NLRP3 oligomerization [27, 128]. This activation pathway requires neither K^+^ efflux, ASC speck formation, pyroptosome formation, nor pyroptosis induction [197, 198] (Fig. 5).

Moreover, the priming to trigger the activated cytokines IL-18 and IL-1β is not always required to induce NLRP3 inflammasome activation. Indeed, because the pro-inflammatory cytokine pro-IL-18, as aforementioned, is already more constitutively expressed in innate cells [141–143, 152] and it was demonstrated that, besides caspases (1/4/5/11) that cleaves pro-IL-18 in NLRP3 canonical activation pathway, other proteases, such as proteinase 3 or neutrophils elastase, can cleave pro-IL-1β into its mature form [200], inducing activation of NLRP3 inflammasome-associated inflammatory signals.

### NLRP3 inflammasome regulation and dysfunction

#### Regulation of NLRP3 inflammasome activation

In normal circumstances, NLRP3 inflammasome activates upon microbial infections and contributes to host defense, helping to clear pathogens through a programed infected cell death mechanism known as pyroptosis. However, as with many other human defense mechanisms, including T- and B-cell adaptive responses, malfunction of NLRP3 inflammasome activation becomes detrimental to host homeostasis. Therefore, it is necessary for the NLRP3 inflammasome to be highly and precisely regulated, to provide adequate immune protection and maintain host health without causing damage to the host tissues and organs.

Specifically, whatever the pathway as described above, activation of NLRP3 inflammasome induces release of mature IL-18 and IL-1β cytokines. Hence, the regulation system consists of preventing the uncontrolled release of IL-18 and IL-1β to maintain inflammatory homeostasis. The mechanisms that regulate NLRP3 inflammasome activation are well described. These mechanisms include post-translational modifications (PTMs) of NLRP3 and NLRP3-interacting molecules that turn on and off the activation of NLRP3 inflammasome.

### Regulation by post-transcriptional modifications

A plethora of studies report that the NLRP3 inflammasome can be activated independently of NLRP3 transcription, suggesting that the priming process has other essential regulatory mechanisms. Deubiquitination and phosphorylation of NLRP3 are the two PTMs that are involved in the regulation of NLRP3 inflammasome activation and inhibition.

Upon priming or in presence of NLRP3 inflammasome stimuli such as LPSs, ubiquitination of the macrophage NLRP3 proteins is inhibited to promote the NLRP3 oligomerization and inflammasome complex formation and NLRP3 proteins remain deubiquitinated during the inflammatory process, until the pathogen is cleared. Inhibition of NLRP3 ubiquitination is mediated by SCFFBXL2 E3 ligase (FBXO3), which targets and senses FBXL2, an endogenous mediator of NALP3 degradation NLRP3 via Trp-73 interaction. In fact, the knockdown of FBXO3, known to interact with ubiquitin, yields in low release of IL-18 and IL-1β [201], involved in inflammatory response. Once the inflammatory process has cleared the infection, to maintain immune homeostasis and avoid detrimental effects, E3 ubiquitin ligase TRIM31 and dopamine directly bind to NLRP3 protein and attenuate NLRP3 inflammasome activation by promoting Lys-48-linked polyubiquitination and proteasomal degradation of oligomerized NLRP3 proteins [202, 203].

Similarly, it has been demonstrated that stimulation through TLR4 by ligand LPSs activates MAPK8 (JUN N-terminal kinase-1 [JNK1]), which directly interacts with human NLRP3 Ser-198 (Ser-194 residue in mice) and induces phosphorylation of NLRP3 protein [204]. Another study [205] demonstrated that, at the Golgi apparatus, protein kinase D (PKD) interacts with human NLRP3 Ser-295 (Ser291 in mouse NLRP3) and phosphorylates NLRP3, promoting NLRP3 oligomerization. Consequently, JNK1- and PKD-mediated NLRP3 phosphorylation induces NLRP3 deubiquitination, facilitates its oligomerization and self-association, and the subsequent inflammasome assembly. However, it has been demonstrated that NLRP3 phosphorylation can also suppress or repress activation of NLRP3 inflammasome. Indeed, PYD has been found to interact directly with human NLRP3 Ser-5 (Ser-3 in mouse NLRP3), which in consequence prevents NLRP3 inflammasome activation [206, 207]. We are tempted to believe that the phosphorylation of NLRP3 protein selectively depends on whether inflammatory needs to be induced or repressed, in presence of absence of NLRP3 stimuli. Therefore, both in resting cells and after inflammatory-associated infection clearance, PYD might selectively interact directly with human NLRP3 Ser-5 (Ser-3 in mouse NLRP3) and repress NLRP3 inflammasome activation. In the same manner, bile acids and prostaglandin E2 induce protein kinase A (PKA)-mediated phosphorylation and thus repress activation of NLRP3 inflammasome [128, 131].

Besides deubiquitination and phosphorylation of NLRP3 protein, another PTMs, including sumoylations have been identified to be involved in regulation of the NLRP3 inflammasome activity [208]. Indeed, upon priming by NLRP3 inflammasome stimuli, the sumoylation of macrophage NLRP3 is repressed by sentrin-specific protease 6 (SENP6) and SENP7, which induces NLRP3 oligomerization and promotes NLRP3 inflammasome complex formation. However, prior to and after NLRP3 activation, NLRP3 is sumoylated by the E3 SUMO protein ligase MUL1 (also known as MAPL), restraining or negatively regulating NLRP3 inflammasome activation.

### Regulation by NLRP3-interacting proteins

As described above, the main proteins that are involved in forming an activated NLRP3 inflammasome complex to induce inflammatory responses include NLRP3, ASC, and the enzyme pro-caspase-1 (Fig. 5). However, it has been found that other intracellular proteins might interact with NLRP3 protein and modulate activity of NLRP3 inflammasome. Specifically, Pyrin-only proteins (POPs, aka PYDC proteins), CARD-only proteins (COPs), chaperone heat shock protein 90 (Hsp90) and its co-chaperone SGT1, guanylate-binding protein 5 (GBP5), RNA-dependent protein kinase (PKR), migration inhibitory factor (MIF), thioredoxin-interacting protein (TXNIP), microtubule-affinity regulating kinase 4 (MARK4), and NEK7, have been reported to activate and/or inhibit activation of NLRP3 inflammasome, based on the need cell [128, 131, 209].

In resting macrophages, Hsp90 with its cofactor SGT1 are found to interact with NLRP3 forming a complex that protects NLRP3 from degradation and keeps it in an inactive form but ready to be sensed by NLRP3 stimuli. In presence of intracellular ROS induced by NLRP3 stimuli, TXNIP and especially NEK7 interact with NLRP3 and induce NLRP3 inflammasome activation. Similarly, in response to ATP, nigericin, and bacteria-associated stimuli, GBP5 and PKR have been reported to prime NLRP inflammasome activation, even though their roles in the activation of NLRP inflammasomes are controversial [195, 196, 210–215].

Reversely, to down-regulate or to negatively regulate NLRP3 inflammatory response after pathogen clearance, it was demonstrated that POP1 and POP2 (among the 4 POPs (POP 1–4)) bind to ASC and inhibit NLRP3-ASC interaction [128]. In this context, it is the last product of the activated NLRP3 inflammasome complex pathway (IL-1β) which has a feedback effect (similar to an allosteric effect) on POP1 and POP2 to downregulate NLRP3 inflammasome activation or prevent its over activation (reviewed in [128, 209]).

#### NLRP3 inflammasome dysfunction and associated diseases

While activation of NLRP3 inflammasome should be associated with host defense against infections and infection relief, its aberrant or improper activation, inactivation and dysfunction during infection, or lack of shutdown after infection release (hyper-activation) are detrimental to health and associated with several health disorders (Fig. 2 and Table 1).

Mutations within NRLP3 inflammasome compounds have been the main causes of dysregulation of NLRP3 inflammasome activation and responsible for inflammatory response-associated diseases. The gain-of-function mutation(s) within Nlrp3 gene have been the first cause of NLRP3 inflammasome dysregulation and associated with inflammatory disorders, one of which being CAPS, a rare condition covering familial cold autoinflammatory syndrome (FCAS, MIM 120100), also known as familial cold urticaria (FCU), Muckle–Wells syndrome (MWS), and neonatal onset multi-systemic inflammatory disease (NOMID) [216]. CAPS is thought to be symptomatically characterized by chronic fever, rashes, inflamed eyes, arthritis, swelling, headaches, deafness and amyloidosis [217]. Indeed, mutations identified within Nlrp3 genes (including CIAS1 that encodes NACHT and LRR [216] and NLRP3-encoding residues adjacent to Ser295 [218]), were associated with aberrant activation of NLRP3 inflammasome, which is the main cause of CAPS.

In Alzheimer’s disease, accumulation of fibrillar peptide amyloid-β after phagocytosis releases cathepsin B that is sensed by NLRP3 (and at a lesser extent NLRP1) and induces activation of NLRP1 and 3 inflammasomes [219], which in turn have been found to worsen Alzheimer’s disease patient conditions [220–223]. To prevent such activation in patients suffering from Alzheimer’s disease, inhibition of NLRP3 and NLRP1 has been found to be promising as it promotes non-phlogistic clearance of amyloid-β and improves cognitive functions [224, 225]. Similarly, NLRP3 inflammasome-associated inflammatory response is not always benefic such as in patients suffering from Parkinson’s disease where NLRP3 inflammasome activation has been associated with neurodegeneration, and that knocking down NLRP3 improve health conditions [105]. A similar detrimental effect of NLRP3 inflammasome activation has been demonstrated in traumatic brain injury that causes neuroinflammation. Indeed, a few hours after traumatic brain injury, NRLP inflammatory mediators, including NLRP3 are upregulated, increasing the activation of inflammation and release of pro-inflammatory cytokines [226]. Studies have demonstrated that patients who have suffered from traumatic brain injury have an increased risk for chronic inflammatory activation in neurons and consequently neurodegenerative diseases [227–229]. These chronic neuro-inflammatory responses are thought to increase hyperphosphorylation of tau protein and amyloid-β, two precursors that worsen Alzheimer’s disease through NLRP3 inflammasome activation.

NLRP3 inflammasome activation and of IL-1β, IL-1 and IL-18 are thought to play a detrimental role in multiple sclerosis (an autoimmune neurodegenerative disorder caused by infiltration of autoreactive T-cells inside the central nervous system through a weakened blood–brain barrier) by facilitating immune cell infiltration and promoting excessive inflammatory response, which in turn aggravates conditions in multiple sclerosis patients [20, 105].

Dysregulation of NLRP3 inflammasome activation, specifically chronic activation of NLRP3 inflammasome, has been involved in pathogenesis of rheumatoid arthritis, gouty arthritis, diabetes and worsening disease conditions. In fact, release of particles in rheumatoid arthritis (pentaxin 3 and its ligand C1q), gout (uric acid crystals), and diabetes significantly over-activates NLRP3 inflammasome [230, 231], which contributes in development of these peripheral inflammatory diseases [232]. Finally, the activation of NLRP inflammasomes has a protective role in different types of cancer; however, its over-activation has been associated with a destructive and promoting role for cancer development [105].

## NLRP6 inflammasomes

### NLRP6 inflammasome and its role in health and innate immunity

The role of NLRP6 to form a cytosolic inflammasome complex and to be involved in innate immunity has long been a debate and non-conclusive. Recent reports have concluded that NLRP6 is a standalone protein forming inflammasome, as they have shown that NLRP6 is able to form an inflammasome complex and cleaves precursor of and release IL-1β and IL-18 during microbial infection [109, 233, 234]. Specifically, NLRP6 was initially shown to activate both caspase-1 and NF-κB. This double activity of NLRP6 inflammasome, unique in NLRP inflammasomes, has been associated with a broad range of physiological functions, including modulation of the host-microbial interface [110, 235, 236], host defense against pathogens [237, 238], and inhibition of carcinogenesis [109] and neuro-inflammation [239]. NLRP6 is one of the major NLRP inflammasomes found in the intestine and liver and was discovered to protect from colitis and ensure homeostasis of intestinal and gut microbiota, and regulate intestinal antiviral innate immunity [235, 236]. Moreover, NLRP6 inflammasome is also found in the kidney tissues and neuronal, lymphocyte, and bone marrow-derives cells [240, 241]. Studies on NLRP6 inflammasome have gained interest just recently because of the significant role NLRP6 inflammasome plays in regulating inflammation and host defenses in specific tissues and organs. In bone marrow-derived macrophages, NLRP6 inflammasome suppresses inflammatory signaling [238]. In goblet cells, the intestinal mucosal epithelial cells that mainly synthesize mucus, NLRP6 inflammasome mediates mucus secretion upon stimulation by TLR ligands or microbiota-associated metabolites, thereby regulating the intestine bacterial population diversity and preventing their abnormal increase or intestine invasiveness by new bacteria [237, 242, 243]. In response to infection by viruses, such as RNA viruses, NLRP6 inflammasome aims to regulate the expression of numerous IFN-stimulated genes through the mitochondrial adaptor protein MAVS [235] (Fig. 2). Relatively newly discovered amongst the NLRP inflammasomes, NLRP6 inflammasome activation mechanism is not fully understood yet; researches are still on going.

### Structural and functional organization of NLRP6 inflammasome

NLRP6 protein, initially known as PYPAF5 protein, has been found to recruit and assemble with ACS protein, caspase-1, or caspase-11 to form an inflammasome complex, mediating proteolysis-based maturation and secretion of IL-8 and IL-1β [109, 234, 244]. NLRP6 inflammasome consists of consists of the NLRP6 protein (the sensor), ASC (the adaptor) and, pro-caspase enzyme (the effector) (Fig. 1). NLRP6 protein is highly expressed in intestinal epithelial goblet cells, where the activated NLRP6 inflammasome complex is responsible for regulating the gut microbiome composition and involved in gastrointestinal inflammatory [235, 236]. NLRP6 protein is also expressed in lungs, liver, and tubular epithelium of kidneys [109, 233, 245, 246]. In buccal cavity cells, especially in gingiva and periodontium cells, NLRP6 inflammasome plays a central role in homeostasis regulation [247, 248]. This distribution of NLRP6 through tissues and cells lies on the multiplicity of NLRP6 transcriptional promotors, which are tissue- or cells-selectively up-regulated. The Human Nlrp6 gene carries three alternative promotors. The first promotor is located in exon 1 (in the 5’ UTR) and modulates expression of NLRP6 in intestine. The second promotor is located in exon 2 (within the PYD domain) and modulates expression of NLRP6 in kidney, liver, lung, neurons, and spleen. The third promotor is located in exon 3 (in the region between PYD and NBD or NACHT) [249]. However, in mice, the Nlrp6 gene carries two tissue-specific promotors; one is located in the exon 1 (in the 185 base-pair of 5’UTR) and promotes expression of NLRP6 in intestinal tissues, and the other is a result of the alternative splicing of exon 1, but located in 1749 base-pair 5’UTR, and promotes expression of NLRP6 in kidney and liver [249, 250].

Like other NLRPs, NLRP6 consists of three domains (Fig. 1): *i*) the PYD, which interacts with ACS, is essential to initiate NLRP6 complex formation [251], *ii*) the NBD (or the NACHT) in the center of the complex followed by *iii*) the LRR domain that is involved in sensing microbial PAMPs and/or DAMPs (review in [245]). Unlike NLRP3 PYD which cannot promote NLRP3 ACS^PYD^ polymerization alone but only complexed with NBD (NACHT), NLRP6 PYD is a stronger nucleator and, in somewhat high concentration, is able to promote NLRP6 ACS^PYD^ polymerization. Specifically, the NLRP6 PYD alone is capable of auto-assembling into filamentous structures followed by large conformational changes and uses PYD-PYD interactions to recruit the ASC adaptor [251]. However, it was shown that the complex formed by PYD-NBD of NLRP6 is stronger in NLRP6 polymerization than the PYD domain alone, which suggests that this assembling process is strengthened by the PYD fused to its NBD (NACHT) domain of NLRP6.

### Molecular mechanism of NLRP6 inflammasome activation

Despite the crucial role played by NLRP6 inflammasome in host defense regulation especially in intestinal microbiota and innate immune signaling in myeloid cells as detailed hereinbefore, its activation molecular mechanism is yet to be fully decrypted, as it has only been recently discovered. Nevertheless, currently available studies have made a non-negligible contribution to exploring and detailing the molecular mechanisms of NLRP6 inflammasome assembly and activation.

**Fig. 6:**
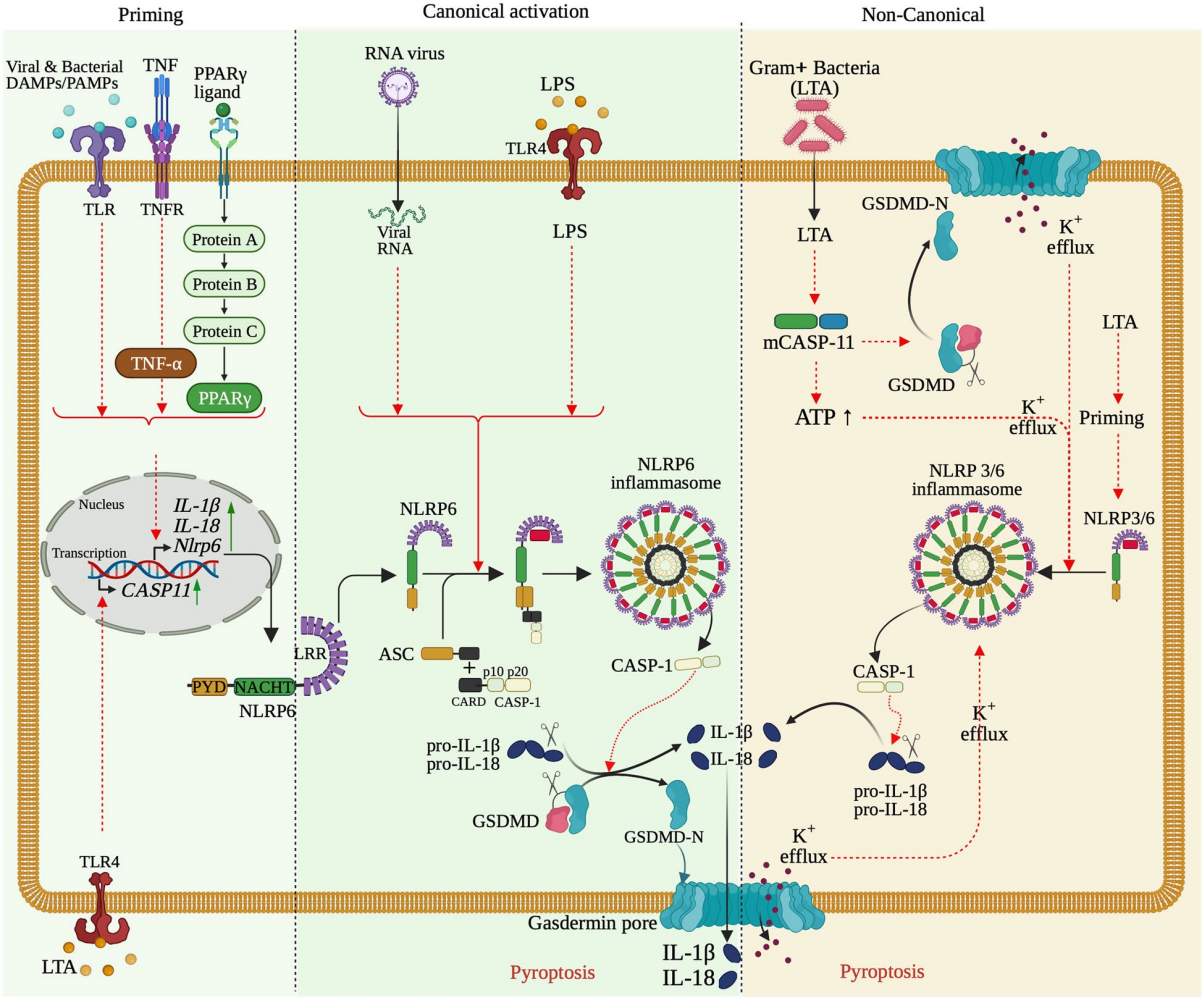
The mechanisms of human NLRP6 inflammasome activation. The activation of NLRP6 inflammasome obey a two-steps mechanism: a priming and an activation. In the priming step, induction of the *Nlrp6* gene transcription and other NLRP6 inflammasome components is triggered by TNF‐α, viral and bacterial PAMPs/DAMPs, and/or the peroxisome proliferator‐activated receptor-γ (PPAR‐γ). Once translated, NLRP6 inflammasome is activated by dsARN from RNA virus and LPS, and occurs through NLRP6 recruitment of ASC and Casp1. The activated NLRP6 inflammasome activates IL-1β, and IL-18 from their respective precursors (pro-IL-1β, and pro-IL-18, respectively) by catalytic cleavage. NLRP6 inflammasome also catalyses digestion of GSDMD into GSDMD-C and GSDMD-N that forms gasdermin membrane pore, which yields to pyroptosis. IL-1*β* and IL-18 cytokines are release out of the cells to promote pro-inflammatory responses. Besides, the NLRP6 protein is also found in the cytosol in low level and is autoinhibited in quiescent cell condition. In this condition, LTA from Gram + bacteria induce an indirect non-canonical activation of the NLRP6 inflammasome. LTA activates caspase-11 (involved in the non-canonical inflammasome activation pathway) which trigger activation of NLRP3/6 inflammasomes, through production of ions flux (specifically K^+^ efflux via GSDMD pores), which in turn activate caspase-1 and release IL-1β and IL-18

From current reports, the activation mechanism of NLRP6 inflammasome is similar to the canonical pathway of NLRP3 activation, described as a two-step model, including a priming step and an activation step. Like with NLRP3 inflammasome activation mechanism, the NLRP6 inflammasome-priming step is triggered by NLRP6 stimuli and lies on induction and transcriptional regulation of the Nlrp6 gene and the expression and oligomerization of NLRP6 protein. The activation step is required to achieve NLRP6 inflammasome assembly, which is followed by inflammatory response characterized by pyroptosis (Fig. 6).

#### Signal 1: priming the NLRP6 inflammasome

The upstream NLRP6 inflammasome stimuli that mainly trigger the up-regulation of Nlrp6 expression include pro-inflammatory signals and metabolites such as TNF‐α or viral and bacterial PAMPs/DAMPs [235, 245, 245, 250] (Fig. 6). Few molecular regulators have been found to prime and induce up-regulation of NLRP6. Upon immune cell exposition to microbial and metabolic NLRP6 stimuli, the peroxisome proliferator‐activated receptor-γ (PPAR‐γ), a transcription factor involved in metabolic regulation [252], and its agonist rosiglitazone [253], induce transcription of Nlrp6 and high expression of NLRP6 protein. More precisely, PPARγ directly binds to the PPAR‐γ-retinoid X receptor-α (PPAR‐γ-RXR-α) at the promoter region of Nlrp6 and induces transcription and expression of NLRP6 [253]. Besides, Roux-en-Y gastric bypass (RYGB) has also been shown to prime NLRP6, through up-regulation of NLRP6 expression upon exposition of intestine cells to microbiota-related metabolites, taurine, and histamine, resulting from intestinal permeability due to obesity [254]. Moreover, as the first priming signal, in interesting study by Hara et al*.* [240] reported that Gram-positive bacteria, such as *Listeria monocytogenes*, induce up-regulation transcription of Nlrp6, expression of both NLRP6 and caspase-11, and then activation of NLRP6 inflammasome. Specifically, the lipoteichoic acid from *L. monocytogenes* induces activation of PPARγ but more specifically up-regulates type 1 interferon (IFN-1) signaling in macrophages, which in turn induces the expression of NLRP6 and caspase-11 [237, 240] (Fig. 6).

#### Signal 2: activating the NLRP6 inflammasome

Once translated, NLRP6 activation requires specific triggers to induce assembly into a typical inflammasome complex, which will activate pro-IL-1β and pro-IL-18 from their respective precursors. These triggers are known as a second signal or signal 2. Amongst the second signals activating NLRP6, lipoteichoic acid (LTA) has been found to serve as a ligand to interact with NLRP6 and activate inflammasome via a signaling cascade of protein recruitment. In fact, LTA binds to the expressed NLRP6 through its LRR domain, promoting ASC poly-oligomerization. Particularly, LTA-based activation of NLRP6 induces the non-canonical NLRP6 inflammasome activation pathway. Indeed, functional NLRP6 activation by LTA activates does not activate caspase-1 directly, but rather does activate caspase-11 [240], which is involved in the non-canonical inflammasome activation pathway. The caspase-11-associated activation of non-canonical pathway triggers activation of NLRP3/6 inflammasomes, through production of ions flux (specifically K^+^ efflux via GSDMD pores), which in turn activate caspase-1 and release IL-1β and IL-18 [250].

Recent reports have highlighted a novel NLRP6 inflammasome activation model, where LTA but specifically viral dsRNA can promote NLRP6 to form a liquid–liquid phase separation (LLPS) which is associated with NLRP6 activation [250]. NLRP6 would form LLPS thanks to its polybasic domain located within the NACHT domain (350–354) and after phase separation, ASC will solidify NLRP6 aggregates and will yield NLRP6 inflammasome activation [250, 255].

NLRP6 inflammasome can also be auto-activated in presence of inflammasome stimuli. As described in a review by Ghimire et al*.* [256] it has been demonstrated that PYD and CARD filaments display prion-like properties that facilitate polymerizations of ASC into filamentous structures leading to activation of ASC-dependent NLRP6 inflammasome [56, 129]. In more detail, ASC-dependent NLRP6 inflammasome activation involves a two nucleation-induced polymerization steps, including *i*) a first ASC^PYD^ filament nucleation by NLR^PYD^ through a PYD-PYD interaction leading to polymerization of ASC, and *ii*) a second nucleation of caspase-1 CARD filaments by the polymerized ASC^CARD^ through a CARD-CARD interaction that directly activates caspase-1, which induces release of IL-1β and IL-18 [256]. Besides, LPSs of Gram-negative bacteria also activate translated NLRP6. *In-*vitro studies reported that LPSs can interact directly with NLRP6, promoting conformational change and subsequently inducing NLRP6 homo-dimerization [257] (Fig. 6).

### NLRP6 inflammasome regulation and dysfunction

#### Regulation of NLRP6 inflammasome activation

Like for NLRP1 and NLRP3, NLRP6 inflammasome activation needs to be highly and timely regulated, as its chronic imbalanced activation, including inactivation when needed or hyper-activation (excessive inflammation), yields several inflammatory and metabolic diseases [107] (Table 1). As previously described, expression of NLRP6 and activation of NLRP6 inflammasome is up-regulated at the transcriptional and post-transcriptional levels. PPAR‐γ is so far though to be one of the first up regulators of Nlrp6 transcription. Besides, in quiescent cells or in absence of ligand activators, NLRP6 inflammasome activation is prevented by the closed conformation of the LRR and NACHT domains of NLR; this inhibition is known as auto-inhibition [256, 258].

Furthermore, recent reports demonstrated that deubiquitinase Cyld plays an important role in preventing uncontrolled and excessive activation of NLRP6 inflammasome. Interestingly, Cyld deubiquinates NLRP6 by destroying the bound to K63 that maintains NLRP6 ubiquitinated, thereby preventing NLRP6 from recruiting ASC [259]. Specific LPSs, including γ-D-glutamyl-meso-diaminopimelic acid (iE‐DAP, an agonist of NOD1), Pam3CysSerLys4 (Pam3CSK4, a synthetic triacylated LPS agonist of TLR1/TLR2), and muramyl dipeptide (MDP) were demonstrated to up-regulate NLPR6 inflammasome activation [243]. Beyond bacterial product, which additionally includes LTA as a strong up-regulator of NLRP6 inflammasome activation together with taurine, histamine, and spermine, ATP-dependent RNA helicase DEAH (Asp‐Glu‐Ala‐His) box helicase 15 (Dhx15) has been thought to be sensed by NLRP6 and enhances activation of NLRP6 inflammasome [235].

After infection clearance, the NLRP6 inflammasome needs to be down-regulated. Interestingly, it has been demonstrated that Nlrp6 transcription and NLRP6 expression are down-regulated by miRNA‐331‐3p after cerebral hemorrhage [260], which stops the inflammatory response and restores cell homeostasis. Likewise, while miR-650 is thought to promote NLRP6 inflammasome-related apoptosis, NLRP6 itself acts as an auto-inhibitor by decreasing the apoptosis increased by the effect of miR-650 through a direct binding of the Nlrp6 3’UTR and miR-650, to restore cell homeostasis after infection clearance [261]. Besides the described regulation mechanism of NLRP6 activation, further studies would provide more details about, and with NLRP6 inflammasome implications in health and disease.

#### NLRP6 inflammasome dysfunction and associated diseases

When not properly regulated, dysregulation of NLRP6 inflammasome activation manifests several diseases, which include familial Mediterranean fever caused by mutations in the pyrin‐coding gene MEFV, or cryopyrin‐associated periodic syndrome, caused by point mutations in Nlrp6 gene [107] (Table 1). Moreover, because NLRP6 has mainly been associated with gut microbiota protection, dysregulation of NLRP activation has been associated with colitis and persistent gut infection [109, 110]. Besides, NLRP6 inflammasome dysfunctions have been associated with adrenomedullin (ADM) loci and male essential hypertension, which suggests that NLRP6 inflammasome and potentially other NLRP inflammasome dysregulation promote pathogenesis of essential hypertension [262].

## NLRP7 inflammasome

### NLRP7 inflammasomes and its role in health and innate immunity

So far, NLRP7 protein has only been described in various human cells and tissues, including lymphocytes, monocytic cells, lung, spleen, thymus, ovaries, and oocytes, but not in rodents’ [263], where it has been found to assemble into an inflammasome complex and induce pyroptosis [264, 265]. However, the exact role of NLRP7 inflammasome in health is currently controversial. On the first hand, NLRP7 is associated with a pro-inflammatory protective function. NLRP7 has been found to sense bacterial lipopeptides (PAMPs) and form an activated inflammasome complex, promoting activation of caspase-1, maturation and release of IL-1β and IL-18, which induce a protective inflammatory response against intracellular bacterial replication [265]. Moreover, the protective role of NLRP7 inflammasome has also been described in ovaries and oocytes, where it promotes embryonic development, though the mechanism modulating this protective role is unknown [263, 266]. On another hand, activation of NLRP7 inflammasome has been associated with an anti-inflammatory role [264, 265]. Specifically, NLRP7 can prevent secretion of IL-1β and inhibit the effector function mediated by the NLRP3 inflammasome [234, 267]. Note that a mutation in Nlrp7 gene found in peripheral blood mononuclear cells from hydatidiform mole patients has been strongly associated with a reduced secretion of IL-1β upon LPS treatment, which was not the case in healthy individual cells. Taken together, it is fair to assume that mutation(s) in NLRP7 might induce NLRP7 inflammasome anti-inflammatory activation response and might be specific to tissues or cells. Complementarily, because the anti-inflammatory response associated with NLRP7 inflammasome might be caused regardless of Nlrp7 mutation, it would be coherent to assume, as suggested by Zeng et al*.* [268], that NLRP7 would negatively regulate inflammation in quiescent cells, while upon stimulation by PAMPs and DAMPs, NLRP7 would promote inflammasome assembly, activation of caspase-1, and release of pro-inflammatory cytokines. Whatever the case is, in-depth studies are required to elucidate the role of NLRP7 inflammasome in inflammatory response.

#### Structural and functional organization of NLRP7 inflammasome

NLRP7 inflammasome has been found to form bona-fide inflammasome complex triggered by acylated bacterial lipopeptides [264, 265]. It consists of the sensor NLRP7, the adaptor ASC, the effector adaptor, and the effector pro-caspase-1 (Fig. 1). NLRP7 senses stimuli (such as ATP) through its NACHT domain to form inflammasome and remain ubiquitinated to regulate its functions [269]. However, its ability to form inflammasome complex remain to be elusive, as several studies report contradictory functions of NLRP7 inflammasome in health and diseases. Overall, the mechanism leading to NLRP7 inflammasome activation remains to be elucidated.

#### NLRP7 inflammasome-associated diseases

Like NLRP3 and NLRP6 inflammasomes, NLRP7 inflammasome activation needs to be highly regulated regardless of its functions (pro- or ant-inflammatory), to insure and maintain the associated protective functions. It has been reported that mutations occurring in Nlrp7 gene are associated with hydatidiform mole, a gestational trophoblastic disease that develops during the early stage of pregnancy and is responsible for a nonviable fetus [111, 266, 270]. Interestingly, these loss-of-function mutations that lead to hydatidiform mole phenotypes are mainly located within the LRR domain of Nlrp7 gene [266], which suggests that LRR play a central role in NLRP7 inflammasome activation. Moreover, it has recently been suggested that a decreased level of NLPR7 protein in trophoblasts disrupts the methylation of DNA and CpG and promotes differentiation of trophoblast lineages, which in turn causes typical trophoblast hypertrophy. This function has only been described for NLRP7 inflammasome and indicates that Nlrp7 is involved in chromatin programming [112].

## Other NLRP inflammasomes

Besides NLRP1, the newly identified CARD8, NLRP3, NLRP6, and NLRP7, known to form bona-fide inflammasomes, several other NLRP family proteins have been reported as sensors in inflammasome complex formation. For instance, NLRP2, NLRP9, NLRP10, and NLRP12 have been found to sense inflammasome stimuli, use ACS as adaptor molecules, and induce release of pro-inflammatory cytokines. However, for some of them, their roles in health are still unclear and their activation mechanisms are to be investigated.

### NLRP2 inflammasome

NLRP2 (aka NALP2, PYPAF2, NBS1, PAN1, and CLR19.9) is expressed in many human cells, including astrocytes [271] and proximal tubular epithelial cells specifically in people suffering from inflammatory diseases [272]. The ability of NLRP2 to form an inflammasome has recently been suggested, though little is known about how NLRP2 specifically mediates to assemble into and form an activated inflammasome. As with all NLRP inflammasome structural organization, the NLRP2 inflammasome is a multiprotein complex composed of the sensor NLRP2, the adaptor ASC, and the caspase-1. It is thought that, like NLRP1, NLRP2 inflammasome would assemble through a direct interaction of NLRP2 with ASC and subsequently with CARD8 domain, which interacts with and regulates caspase-1 activation [273–275]. In human astrocytes, the DAMP such as ATP mediates NLRP2 inflammasome activation, leading to the processing of inflammatory caspase-1 and interleukin-1β (IL-1β). In addition, it was found that NLRP2 could interact with the P2X7 receptor and the pannexin-1 channel, leading to NLRP2 inflammasome activation [271]. Moreover, NLRP2 positively up-regulates pro-fibrotic mediator expression and NF-κB activation, by modulating the p65 NF-κB phosphorylation, but down-regulates that expression of several interferon-inducible genes. However, overexpression of NLRP2 in proximal tubular epithelial cells hampers the apoptotic reaction [272]. Besides, kynurenine, a tryptophan metabolite produced as a biomarker in the immune dysfunction of depression, was recently shown to activate NLRP2 inflammasome in astrocytes. An increased level of kynurenine in the mouse hippocampus together with the presence of ATP is associated with NLRP2 inflammasome activation, which is characterized by expression of caspase-1 and release of IL-1β. More interestingly, a treatment with kynurenine promotes the translocation of NF-κB to the nucleus and its binding to NLRP2 promotor, which subsequently induces an increased NLRP2 transcription, modulating inflammasome activation [276]. This recent study by Zhang et al*.* [276] evidenced that NLRP2 inflammasome plays an important role in depressive behavioral, as it participates in inflammatory immune response. Knocking-out kynurenine and/or NLRP2 hampers inflammation and restores homeostasis, which suggests that drugs targeting kynurenine or NLRP2 in depression-like behaviors would relieve from these states. NLRP2 is also expressed in human brain vascular pericytes, together with NLRP1, NLRP3, NLRP5, NLRP9, and NLRP10. In these brain cells, NLRP2 inflammasome activation occurs through the non-canonical activation pathway triggered by intracellular LPS or *E. coli.* Bacteria [277].

### NLRP9 inflammasome

Similar to NLRP1, which is represented by only one gene in humans (hNLRP1), but four paralogues in rodents (mNLRP1*a-d*), NLRP9 is represented by only one gene in humans (hNLRP9) but three isoforms in rodents (mNLRP1*a-c*) [25] and are mainly expressed in reproductive organs [278]. Specifically expressed in human, murine, and bovine oocysts, ovaries, and testes, NLRP9 expression has been associated with preimplantation and development of embryos, and lack of all mNLRP9 isoforms [279, 280] (not a single one [281, 282]) in mice hampers embryonic preimplantation and development.

While its role in reproductive cells is somewhat elaborated, NLRP9 is one of the less- and the last characterized NLRP family proteins in terms of protein-forming inflammasome. Thus, the role of NLRP9 in infectious and inflammatory diseases is still elusive. Recent studies have reported that NLRP9 could initiate assembly into a protective activated inflammasome involved in host immune defense against proliferation of infectious diseases, especially in the intestine. Specifically, except in reproductive cells, mNLRP9*b* (not mNLRP9*a* or mNLRP*b*) was also found to be highly expressed in the ileum, where it was associated with caspase-1 cleavage and IL-18 release upon rotavirus infection, as mNLRP9*b*-depleted mice showed elevated viral load and severe pathogenesis compared to wild-type mice harboring mNLRP9*b* [283]. This suggests that NLRP9 can potentially form a bona-fide inflammasome that triggers a protective inflammatory response against pathogens.

In regards to what precedes, conclusions on the molecular and structural mechanisms of NLRP9 activation would be based on mNLRP9*b* investigations, as there are few or no studies on NLRP9 inflammasome mechanisms of assembly. Yet, how NLRP9 inflammasome assembles remains largely unknown. In their study, Zhu et al*.* [283] demonstrated that mNLRP9*b* serves as a sensor for rotavirus infection to trigger NLRP9 inflammasome formation. Specifically, mNLRP*b* indirectly senses rotavirus RNA through an intermediate binding with DExH-box helicase (DHX)9, an RNA helicase that has a high binding affinity with dsRNA, here rotavirus, and forms a complex. In absence of DHX9 in experimental challenge cells with rotavirus, low production of IL-18 together with resistance to pyroptosis were observed. It is necessary to note that DHX9 is unable to bind host RNA, which could trigger NLRP9 inflammasome-associated auto-inflammatory response but specifically recognizes viral dsRNA, via a mechanism that is still unknown [284]. Moreover, which domain of NLRP9 that binds to DHX9 is also unrevealed. Moreover, how the NLRP9-DXH9-RNA complex recruits ASC and other monomeric subunits necessary for assembly or how the formed complex induces activation of NLRP9 inflammasome, which protects against rotavirus needs to be elucidated. Furthermore, as it is shown for rotavirus in intestine, more studies are needed to explore the NLRP9 effect in other cells and organs.

Conversely, while NLRP9 inflammasome could protect against rotavirus in intestine, another study has demonstrated that mNLRP9*b* inflammasome is involved in enhancement of acute lung injury [285]. Indeed, wild-type mice carrying mNLRP*b* gene showed a higher neutrophilic inflammatory response and a decreased survival rate compared with mNLRP9*b*-depleted mice, which suggests that NLRP9 inflammasome activation, like other NLRP inflammasomes, can be detrimental to health. More interestingly, as recently reviewed [286], besides lung injury [285], NLRP9 inflammasome is involved in the occurrence or the enhancement of many other diseases, including chronic childhood arthritis (systemic-onset juvenile idiopathic arthritis [287]), multiple sclerosis [288], Alzheimer’s disease [289], urothelial carcinoma [290], and *Helicobacter pylori*-associated infection [291]. Note that these last cited NLRP9 inflammasome-associated diseases are related to a mutation in NLPR9 gene that may cause a dysfunction of NLRP inflammasome and its aberrant activation.

### NLRP10 inflammasome

Also known as NOD8, PAN5, or PYNOD, NLRP10 protein was described for the first time by Wang et al*.* [292] and has been found in rodent immune and human cells, including epithelial cells, keratinocytes, macrophages, dendritic, and T cells [246]. However, the expression of NLRP10 in each of these cells and environment seems different and associated with different variable, but contradictory functions. In fact, NLRP10 is the only protein from NLRP protein family that does not have the same structural features as other NLRP proteins known to form inflammasome complex, as it lacks the LRR domain involved in homotypic CARD-CARD domain interactions required to recruit the enzyme pro-caspase-1 for a typical inflammasome complex assembly. Thus, NLRP10 protein only consists of PYD and NBD (NACHT) domains. This characteristic suggests that NLRP10 might have an NLRP10 inflammasome-independent function, as NLRP10 protein may not act like a signal sensor of PAMPs and DAMPs and be involved in forming inflammasome, but rather like a probable regulator or an adaptor [293]. In other words, NLRP10 looks more like a regulator rather than an inflammasome-associated sensor, and may not form a bona-fide inflammasome, unless another domain is involved in recruiting pro-caspase-1 to allow assembly into an active inflammasome with effect on caspase maturation and interleukin release. Notably, Lech et al*.* [246] and Imamura et al*.* [294] demonstrated the anti-inflammatory and inflammasome-independent function of NLRP10 in innate immunity. They showed that NLRP10 protein negatively regulates other inflammasome activation and inflammasome-associated cell death. Specifically, they described that NLRP10 inhibits ASC-mediated NF-κB activation and prevents the release of IL-1β by hampering the caspase-1-mediated maturation of IL-1β. These activities have been also shown in other reports and are attributed to the NACHT domain (and to a lesser extent PYD) of NLRP10, which interacts with caspase-1 through ASC of other inflammasomes [292, 293].

However, these findings may not result in a general conclusion because, it has also been surprisingly and contrarily shown that in other cells different from the human epithelial cells used in the above description, NLRP10 may have an inflammasome-dependent activity, meaning NLRP10 is able to form an inflammasome. Notably, while the negative regulation of NLRP10 was shown by others, reporting inhibition of NLRP3-associated normal canonical activation and IL-1β production in mouse DCs carrying NLRP10 [294], the presence of NLRP10 in transgenic mouse macrophages has no negative effects in ASC aggregate formation, nor does it have negative regulation in caspase-1-mediated maturation of IL-1β. Also, recent studies by Prochnicki et al*.* [295] and Zheng et al*.* [296]have shown that NLRP10 can assemble into a bona-fide inflammasome in differentiated human keratinocytes and is involved in monitoring mitochondrial integrity in an mtDNA-independent manner, as 3M3-FBS triggers NLRP10 inflammasome activation via mitochondrial disruption. This suggests that NLRP10 expression seems to depend on cell-type and its function is cellular environment- and signaling pathways-dependent [296–298]. Therefore, more investigations are required for a better understanding of the NLRP3 inflammasome-dependent and independent functions in innate immunity and whether, or not, NLRP10 forms a functional inflammasome. Such studies are relevant to facilitating new innate immune anti-inflammatory interventional strategies [296].

### NLRP12 inflammasome

NLRP12 protein (aka Monarch-1 or Pypaf7) is mainly expressed in immune cells, including bone marrow-derived dendritic cells, granulocytes, macrophages, and neutrophils [299, 300]. Like NLRP10, NLRP12 was described as a negative regulator for normal canonical activation and IL-1β secretion in activated B-cell signaling through interaction with IRAK1 that prevents its accumulation. Similarly, NLRP12 down-regulates non-canonical activation through interaction with TRAF3 that promotes the degradation of NF-κB-inducing kinase [301–303]. In hematopoietic and non-hematopoietic stem cell subsets, NLRP12 has been found to act like an inflammatory response modulator [301]. In addition, it has been found that NLRP12, together with NLRP10, can negatively regulate adaptive immunity [304].

Besides being an immune response regulator and like other NLRP family proteins (except NLRP10), NLRP12 has been found to inflammasome-forming component. This role of NLRP12 in forming inflammasome and the inflammasome-associated function was evidenced in part from infection with Yersinia pestis, in which NLRP12 inflammasome induced activation of caspase-1 and release of pro-inflammatory cytokines, including IL-1β and IL-18, and was associated with protection against *Yersinia pestis* infection [305]. However, in the *in-vivo* infection model, NLRP12 and NLRP3 inflammasomes are concomitantly required to provide an anti-infectious resistance against *Yersinia pestis*, which suggests that in some infection setups, activation of divers NLRP family proteins or NLRP inflammasomes might provide an optimal protection again microbial antigens [306].

NLRP12 was amongst the first NLRP family proteins to be described together with NLRP1 and NLRP3, and displays structural similarities with NLRP3 [299, 300]. However, the structural and functional organization of NLRP12 inflammasome needs to be studied. Even though NLRP12 inflammasome activation mechanism is still unknown, its activation process should be highly regulated to ensure health and homeostasis. Indeed, dysregulation of NLRP12 inflammasome activation, in part caused by numerous identified mutations (above 20 mutation types [307, 308]), has been associated with health disorders and systemic inflammatory diseases [307–309]. Patients displaying CAPS-associated symptoms have been shown to carry a set of NLRP12 but not always NLRP3 mutations, considered to dysregulate NLRP12 inflammasome activation and trigger the NLRP12-associated auto-inflammatory diseases (NLRP12-AID) [307–309]. However, it is important to note that certain of these NLRP12 mutations do not always compromise the protective pro-inflammatory effect of NLRP12 inflammasome but rather promote a gain-of-function, as they are associated with increased caspase activation and enhanced IL-1β secretion. Therefore, these conflicting roles of NLRP12 [306], including *i)* the NLRP12-assocciated protective pro-inflammatory function, *ii)* the NLRP12 mutation-associated diseases triggering function, and *iii)* the NLRP12 negative regulation role described above might have rendered initial attempts for anti-IL-1 therapy difficult and unsuccessful, and may contribute to explain mechanisms underlying resistance to anti-IL-1 therapy observed in patients with CAPS [310]. Thus, to provide a successful therapy against such a scenario, further in-depth studies are highly needed.

## Therapeutic strategies against pathogenic NLRP inflammasomes

As previously described, aberrant NLRP inflammasome activation and gain-of-function mutations have been associated with the development and enhancement of numerous metabolic, auto-inflammatory, autoimmune, and neurodegenerative diseases. The currently used immunosuppressive and anti-inflammatory treatment, which include cyclosporine, steroids, methotrexate, and general anti-TNF-a therapy allows to treat severe cases of inflammatory diseases [304, 311]. However, a challenge rises in such a way that the immunosuppressive and anti-inflammatory treatment may hamper activation of a normal protective immune response that is not associated with disease-specific pathological mechanisms. Thus, to overcome this challenge, it is suggested to thoroughly understand the difference between activation mechanisms of the proper induction of protective immune response and that is specifically leading to diseases, as this would allow development of aberrant inflammasome-specific drugs that could not hamper immune response induced to clear infection [304].

Fortunately, to pave this way, promising therapeutics that specifically and selectively inhibit aberrant NLRP inflammasome activation in inflammatory diseases have been developed and proven effective. As summarized in Table 2, these therapeutics mainly target and inhibit NLRP inflammasome products, including IL-1β and IL-18, and hamper the NLRP inflammasome activation ability of sensor stimuli [312]. For example, the inhibitors in CAPS that inhibit downstream pro-inflammatory cytokine also contribute to reducing the pathogenic effect of CAPS by blocking inflammasome-independent but CAPS-dependent pyroptosis released DAMPs that are produced to induce more CAPS-related pathological inflammation [313]. The best example of inhibitors that play such roles includes canakinumab and anakinra, a monoclonal anti-IL-1β antibody and a recombinant IL-1 receptor antagonist (IL-1RA), respectively. Canakinumab and IL-1RA anakinra are currently approved for treating certain forms of arthritis, CAPS, and Mediterranean fever, and they are effective in reducing cardiovascular events in atherosclerosis patients [314]. As highlighted by Bulte et al*.* [304], these inhibitors may still hamper induced protective immunity upon infection.

**Table 2:** Pharmacological inhibitors of NLRP inflammasome activation

	**Therapeutics (candidates)**	**Inhibit(s):**	**Diseases treated**	**Ref.**
**NLRP3 inflammasome**	Glyburide	ATP, nigericin, and NLRP3-associated IAPP and IL-1β production	TD2	[315, 316]
	16,673–34-0	Glyburide derivate substrate	AMI	[317]
	RRX-001**		SCLC and severe oral blistering or mucositis	[318, 319]
	ZYIL1*	NLRP3 oligomerization, IL-1β and IL-18	CAPS	[320]
	JC124	NLPR3, ASC, caspase-1, and pro-IL-1β production	Protect from TBI enhancement	[317]
	FC11A-2	IL-1β, IL-18 production and caspase-1 activation	Colitis	[321]
	NRTIs	P2X7 signaling	Retrovirus Infection, auto-inflammatory and autoimmune diseases	[322]
	VX-740*, VX-765*	CASP-1 activation and pro-IL-1β/IL-18 production	RA, OA	[317, 323]
	MCC950*	IL-1β and IL-18 production	Aberrant NLRP3 activation-based inflammatory diseases	[317, 323]
	MNS	ATPase activity by binding to NLRP3 LRR and NACTH		[144, 324, 325]
	CY-09 (Glitazone)*	ATPase activity and NLRP3 activation	Depression-like behavior, Alzheimer’s disease	[70, 317, 323, 326]
	Tranilast*	NLRP3-ASC interaction	Homologous passive anaphylaxis	[317, 323]
	OLT1177*	IL-1β and IL-6 production	Degenerative arthritis, gout	[323, 327]
	Oridonin*	NF-κB pathway and IL-1β and IL-18 production Abolish NLRP3-NEK7 interaction	Neuro-inflammation, sepsis, colitis, TD2, peritonitis, gouty arthritis	[317, 323, 328]
	Selnoflast*	IL-1β production	CAPS, ulcerative arthritis, IBD	[329]
	CRID3	ASC oligomerisation		[330]
	Auranofin	NLRP3 IL-1β production		[331]
	ILG	ASC oligomerization, IL-1β and caspase-1 production	HFD-induced obesity and macrovesicular steatosis, and adipose tissue inflammation	[332]
	25-HC	IFN signaling and IL-1	Septic shock, exacerbated experimental autoimmune encephalomyelitis, and bacteremia	[304]
	BHB	IL-1β and IL-18 production, and K^+^ efflux	FCAS, MWS	[333]
	IL-1RA anakinra**	Downstream effect of IL-1	CAPS, arthritis, MEFV	[97, 334]
	Canakinumab**	IL-1β NmAbs	CAPS, arthritis, MEFV	[97, 334]
	Tranilast	NLRP3 oligomerization	NLRP3 inflammasome-associated diseases	[317, 335, 336]
	DFV890*	NLRP3 inflammasome activation	CAPS, knee osteoarthritis, COVID-19, and pneumonia	[157]
	Ibrutinib*	NLRP3 Phosphorylation	Colitis	[323, 337]
	VI-16*	TXNIP-dependent NLRP3 activation	Colitis	[323, 338]
	INF39*	ATPase activity of NLRP3		[323, 339]
	Disulfiram**	Formation of GSDMD pore and IL-1β release		[323, 340, 341]
	IZD174*, IZD334*		CAPS	[157]
	VTX2735	IL-1β and hsCRP	Inflammatory diseases, CAPS	Link
	NT-0796 / NT-0249*	hsCRP	Inflammatory diseases	[342]
	HT-6184*	NEK7 and NLRP3 pathways		[343, 344]
	Rilonacept	Downstream effect of IL-1	CAPS	[97, 334]
**NLRP3 and NLRP1**	Parthenolide*	CASP-1 activation; NLRP3 ATPase activity		[345]
	Bay 11–708	NF-κB pathway		[317, 345]
	CPG 15d-PGJ2	CASP-1 activation		[346]
	RKIP	CASP-1 activation and IL-1β secretion	Gouty arthritis and T2D	[347]
	IVIg**		Ischemic stroke	[348]
	ADS032	IL-1β and TNF-α	Pulmonary inflammation	[349]
	Arsenic trioxide	CASP-1 activation and IL-1β secretion		[350]
	Sodium arsenite	CASP-1 activation and IL-1β secretion		[350]

*Under pre-clinical investigation, clinical trial phase 1, 2, or 3

**FDA pre-approved or approved for clinical use. CAPS, cryopyrin-associated periodic syndromes. TD2, Type 2 diabetes. *IAPP* Islet amyloid polypeptide. *mAbs* monoclonal antibodies*. NmAbs* neutralizing monoclonal antibodies*. NRTIs* nucleoside reverse transcriptase inhibitors*. 25-HC* 25-Hydroxycholesterol*. CPG* cyclopentenone prostaglandin*. TBI* traumatic brain injury*. AMI* Acute myocardial infarction. *SCLC* small cell lung cancer. *RA* rheumatoid arthritis*. OA* osteoarthritis. *CRID3* cytokine release inhibitory drug. *BHB* β-hydroxybutirate*. FCAS* familial cold autoinflammatory syndrome*. MWS* Muckle-Wells syndrome*. MNS* 3,4-methylenedioxy-β-nitrostyrene. *ILG* isoliquiritigenin. *IBD* inflammatory bowel disease*. hsCRP,* high sensitivity C-reactive protein. HFD, hand, foot, and mouth disease. *RKIP* Raf kinase inhibitor protein. *IVIg* intravenous immunoglobulin

Besides, recent research discovered and characterized many inflammasome inhibitors that directly target NLPR genes or NLRP inflammasome complex-associated components, blocking their oligomerization, the further inflammasome activation pathway, and the subsequent release of pro-inflammatory cytokines. For instance, the 3,4-methylenedioxy-β-nitrostyrene (MNS) that has been demonstrated to treat inflammasome activation-induced inflammatory bowel disease (IBD), prevents NLRP3-mediated ASC speck formation and oligomerization and inhibits NLRP3 ATPase activity by binding to NLRP3 LRR and NLRP3 NACTH [324, 325]. Tranilast, another direct NLRP3 inhibitor, inhibits NLRP3 oligomerization by directly binding to NLRP3 NACHT domain and prevents NLRP3 inflammasome assembly [317, 335, 336]. Moreover, VI-16 blocks oligomerization of NLRP3 and activation of TXNIP-dependent NLRP3 inflammasome. Table 2 shows other example inhibitors that directly target NLRPs or NLRPs-associated complexes.. These NLRP inflammasome inhibitors tend to be the best against pathogenic inflammatory responses because, unlike the previous, they inhibit the formation of inflammasome complex, so blocking any downstream activity. Drugs that inhibit NLRP inflammasome products would not prevent inflammasome complex formation. Therefore, treatment with such inhibitors would be a long-term process and difficult to stop, as stopping it would still lead to production of downstream pro-inflammatory cytokines by activated inflammasomes, thus compromising the health state. This later might, however not be observed when treating with inhibitors that directly target NLPRs or NLRP components. In fact, we hypothesized that, besides mutations-based aberrant inflammation, blocking NLRP oligomerization for further aberrant inflammasome complex activation would help the inflammasome regulation system to restore proper regulation of NLRP inflammasome activation. It would be therefore suitable to study whether short-term treatment with direct NLRP inflammasome inhibitors permanently restores cellular homeostasis and ameliorates health conditions in aberrant inflammasome activation-associated diseases.

The way to deal with improper NLRP inflammasome activation and the associated pathological conditions is still long. Note that almost, if not all current NLRP inflammasome inhibitors considered under clinical trials target NLRP3 inflammasome (Table 2 and review in [304]), unless the intravenous immunoglobulin (IVIg) which is an FDA-approved drug against NLRP1 and NLRP3 inflammasome activation [348]. Moreover, as presented in Table 2, most of the discovered or developed inhibitors of NLRP inflammasome activation are only towards NLRP3 inflammasome, but not other described NLRP inflammasomes. This suggests that besides the promising potential to inhibit aberrant activation of NLRP3 inflammasome, considerable progress is required, especially in developing other NLRP inflammasome-specific drugs than those targeting NLRP3 inflammasome [351].

## Concluding remarks and future perspectives

Among the NLRP inflammasomes, NLRP3 inflammasome is the most highly described, with its molecular mechanism of assembly and activation well enough elaborated so far. Its role in health preservation and occurrence of auto-inflammatory, autoimmune, and neurological diseases and aggravation of these conditions is also well described. It is noteworthy that most studies in developing pharmacological inhibitors to treat pathological inflammasome activation are mainly directed against NLRP3 inflammasome, for which promising drugs against NLRP3 inflammasome-associated diseases are in advanced stages of clinical trial. Although encouraging, as these studies/results allow management of NLRP3 inflammasome activation and the associated diseases, huge effort is still required for both understanding of NLRP3 inflammasome mechanisms of modulation and other NLRP inflammasomes mechanisms of activation and modulation, and for the development of specific inflammasome therapeutic inhibitors.

Many parameters have hampered the studies on the mechanism of assembly of certain inflammasomes, such as NLRP1 and NLRP9 inflammasomes. The molecular mechanisms of NLRP1 assembly and activation were poorly characterized because of the lack of common activators in human and mouse models, and because of the inherent structural differences in hNLRP1 and mNLRP1s [25, 31–34]. Current reports have revealed numerous perturbations and molecular entities related to NLRP1 biology (e.g., dsRNA, viral 3C proteases and reductive stress). Notably, NLRP1 stimuli can be divided into two distinct groups based on pathogens signals and danger signals. One group is pathogen signals which contain viral 3C proteases, viral dsRNA, KSHV-encoded ORF45, lethal anthrax toxin, IpaH7.8 of *Shigella flexneri* and *T. gondii* infections. UVB, O_3_, ATP, peptide accumulation, metabolic inhibitors, and reductive and ribotoxic stress belong to the other group of danger signals. Interestingly, the wide variety of agonists has unraveled the pivotal role of NLRP1 inflammasome in cellular homeostasis and host defense responses.

Furthermore, although there are some unresolved mysteries regarding NLRP inflammasome activation, recent studies have provided much about NLRP1, NLRP6, and NLRP7 receptors that can detect diverse pathogens or dangerous activities. Yet, several questions arise to understand the molecular mechanisms of NLRP inflammasome activation. Indeed, regarding NLRP1, one interesting question raised by these observations includes how dsRNA triggers a conformational shift of the N-terminal region of NLRP1 to destabilize the ternary complex remains elusive. Another outstanding question consists of how DPP8/9 inhibitors precisely affect the CARD8-DPP9 ternary complex to activate the NLRP1 inflammasome in cells. Although keratinocytes and AML (Acute Myeloid Leukemia) cells express NLRP1 and CARD8, the molecular mechanism of how VbP activates NLRP1 inflammasome in keratinocytes, whereas CARD8 sense VbP in AML cells, remains unknown. Moreover, understanding how some NLRP inflammasomes are activated and modulated, post-activation mechanisms that trigger pyroptosis might serve as therapeutic targets to modulate or inhibit pathologic NLRP inflammasome activation. Also, it has been thought that expression of NLRPs is associated with either pathologic or benefic effect/response in health or disease based on the tissue; thus more studies are needed to understand what drives these NLRP cell-specific differences of function.

As for NLRP3 activation pathways, studies characterizing the upstream and downstream pathways of NLRP1, NLRP6, NLRP6, and NLRP7 inflammasome activation have suggested important roles in human diseases (Fig. 2 and Table 1). Because the aberrant activation of NLRP inflammasomes is associated with several auto-inflammatory diseases, and their inhibition could be a useful pharmacological approach for managing chronic inflammatory disorders. Many studies have demonstrated that potential NLRP1 inhibitors significantly prevent NLRP1 inflammasome complex formation [351]. In addition, as presented in Table 2, the blockade of NLRP inflammasome downstream signaling, such as the IL-1β receptor, could be a suitable pharmacological approach for treating immune-mediated inflammatory diseases [352].

In the effort to develop therapeutics against aberrant activation of NLRP inflammasomes, it is important to note that most of the current therapeutic inhibitors (Table 2) do not directly target NLRP inflammasome components, but are specific to the final products of inflammasomes such as IL-1β, IL-18, NF-kB signaling, and so on. Thus, we are tempted to suggest that more effort should be put into developing therapeutics that target NLRP inflammasome components. Targeting NLPR inflammasome components, if possible, would be more beneficial because it would reduce the random blockade of normal production of IL-1β and IL-18 for example, which are not always harmful or induced by NLRP inflammasome activation. In addition, such targets could or may be directed to mutant NLRPs that lead to aberrant inflammasome activation, and therefore would solve the question of the limit between the harmful and the beneficial inflammasome activation and how to react accordingly.

Overall, these recent advances in our understanding of the mechanisms of NLRP inflammasome activation offer insight into inflammasome assembly and signaling. Further exploration is needed to gain insight into the complex activation mechanism of other NLRP inflammasomes and how their dysfunction is associated with human diseases.

## Data Availability

Not applicable.
